# Long-read based assembly and synteny analysis of a reference *Drosophila subobscura* genome reveals signatures of structural evolution driven by inversions recombination-suppression effects

**DOI:** 10.1186/s12864-019-5590-8

**Published:** 2019-03-18

**Authors:** Charikleia Karageorgiou, Víctor Gámez-Visairas, Rosa Tarrío, Francisco Rodríguez-Trelles

**Affiliations:** Grup de Genòmica, Bioinformàtica i Biologia Evolutiva (GGBE), Departament de Genètica i de Microbiologia, Universitat Autonòma de Barcelona, Bellaterra, Barcelona, Spain

**Keywords:** Genome structure evolution, Inversion originating mechanisms, Inversion fixation and polymorphism, Spatiotemporally fluctuating selection, Adaptation, Global change

## Abstract

**Background:**

*Drosophila subobscura* has long been a central model in evolutionary genetics. Presently, its use is hindered by the lack of a reference genome. To bridge this gap, here we used PacBio long-read technology, together with the available wealth of genetic marker information, to assemble and annotate a high-quality nuclear and complete mitochondrial genome for the species. With the obtained assembly, we performed the first synteny analysis of genome structure evolution in the *subobscura* subgroup.

**Results:**

We generated a highly-contiguous ~ 129 Mb-long nuclear genome, consisting of six pseudochromosomes corresponding to the six chromosomes of a female haploid set, and a complete 15,764 bp-long mitogenome, and provide an account of their numbers and distributions of codifying and repetitive content. All 12 identified paracentric inversion differences in the *subobscura* subgroup would have originated by chromosomal breakage and repair, with some associated duplications, but no evidence of direct gene disruptions by the breakpoints. Between lineages, inversion fixation rates were 10 times higher in continental *D. subobscura* than in the two small oceanic-island endemics *D. guanche* and *D. madeirensis*. Within *D. subobscura*, we found contrasting ratios of chromosomal divergence to polymorphism between the A sex chromosome and the autosomes.

**Conclusions:**

We present the first high-quality, long-read sequencing of a *D. subobscura* genome. Our findings generally support genome structure evolution in this species being driven indirectly, through the inversions’ recombination-suppression effects in maintaining sets of adaptive alleles together in the face of gene flow. The resources developed will serve to further establish the *subobscura* subgroup as model for comparative genomics and evolutionary indicator of global change.

**Electronic supplementary material:**

The online version of this article (10.1186/s12864-019-5590-8) contains supplementary material, which is available to authorized users.

## Background

*Drosophila subobscura* Collin [1] is a fruitfly species of the *obscura* group of the subgenus *Sophophora* endemic to, and common in Europe and the western Palearctic, where it spans over thirty latitudinal degrees commonly associated to forest fringes, from sea level to the timber line [2]. The species was found to be unusual among *Drosophila* because it is entirely monandrous [3–5], does not mate in the absence of light [6, 7], and does not produce courtship-song by wing vibration [8].

The rise of *D. subobscura* to its current status as model organism for biological research owes to a long-held effort to understand the genetics and evolutionary biology of the species [9]. Early investigations on its salivary gland nucleus revealed that it has the ancestral *Drosophila* karyotype of a small dot and five large acrocentric rods, does not show a chromocenter [10] and, especially, shows extraordinary levels of chromosomal polymorphism caused by large, cytologically visible paracentric inversions segregating on all five rods. Elaboration of detailed polytene drawings [11, 12] and photomaps [13–15] greatly facilitated the study of the inversions, and paved the way for subsequent development of the over 600 linkage [16] and cytologically mapped genetic markers presently available, which cover most of the euchromatic genome [17, 18].

Besides nuclear genetics studies, obtention of the first restriction, and at present the only map available for the *D. subobscura* mitogenome [19] allowed to identify an intriguing geographical pattern of variation with two major mitotypes, named I and II, that segregate at nearly equal frequencies in most populations, and which have associated measurable differences in fitness-related traits [20, 21].

The discovery of the two Macaronesian island-endemic species *D. guanche* Monclús [22], from the Canarian archipelago, and *D. madeirensis* Monclús [23], from Madeira allowed new possibilities for comparison. Together with *D. subobscura*, they form the *subobscura* subgroup [24]. The three species are isolated reproductively from each other, except for *D. madeirensis* and *D. subobscura* [25, 26], which are capable of limited gene exchange in collinear genomic regions not affected by inversions [27]. Hybrid males show extra sex combs, among other anomalies whose genetic basis and role in species formation has only begun to be elucidated [28–30]. Interestingly, the two island endemics show differences in gene ordering between them, and with respect to *D. subobscura*, but are thought to be monomorphic for inversions.

Of the various features of the *D. subobscura* model, its rich inversion polymorphism has received special attention [31]. In total, more than 65 inversions have been identified, which range in length from ~ 1 Mb (e.g. inversion E_20_) to as long as ~ 11 Mb (O_7_). They include both simple and multiple overlapping inversions on the same chromosome, which appear strongly associated into about 90 different chromosomal rearrangements [9, 32]. All combined, structurally segregating regions represent approximately 83% of the species genome. The inversions are nonrandom as to their lengths and distribution of breakpoints along the chromosomes, with cytological evidence of multiply reused breakpoints in 26 cases (~ 20% [9]). Recently, breakpoint nucleotide sequences were determined for nine polymorphic inversions using in situ hybridization and chromosome walking methods, which found one case of breakpoint reuse, and overall supported a mechanism of inversion formation through chromosomal breakage and repair by non-homologous end joining, rather than through ectopic recombination [33, 34].

Inspired by the work of Dobzhansky et alia on natural populations of its Nearctic sister basal within the *obscura* group *D. pseudoobscura*, research on *D. subobscura* found the inversion frequencies in all major chromosomes to be highly structured according to both spatial and temporal environmental gradients. Specifically, chromosomal polymorphisms vary geographically between cold and warm climates [35], with genomewide warm climate inversion frequencies peaking in summer and dropping in winter repeatedly every year (and the reciprocal for the cold climate arrangements) [36]. The introduction, rapid spread, and successful establishment of *D. subobscura* throughout the southern Neotropical [37] and western Nearctic [38] regions, from few colonizers [39], in contemporary time [40] attested for the high dispersal ability and potential for local adaptation of the species [41]. The establishment of latitudinal patterns of the same sign across three separate territories [42] which, additionally, stood in contrast with the uniformity found for neutral nucleotide markers [39], further corroborated the adaptive significance of the chromosomal polymorphisms. On top of these patterns, southernmost populations of the species were found segregating for a sex-ratio distorting drive arrangement, whose carrier males have offspring consisting of only or mainly females [43, 44]. The realization that the frequencies of cold climate karyotypes are declining with the globally rising temperatures [45–47] expanded the interest on the species as indicator of evolutionary effects of contemporary global-warming [48–50]. In fact, the standing inversion variation, maintained by the spatiotemporally fluctuating thermal environment allowed a rapid genomewide evolutionary response in a time scale as short as “few days” during a sudden heatwave [51].

Although the recombination-suppression effects of inversions may not suffice to suppress gene flow in the inverted regions entirely [52, 53], it is strong enough to cause nucleotide variation in *D. subobscura* to be extensively structured in regions affected by the rearrangements [54], and to allow evolution of genomic islands of concerted evolution of ecologically-relevant gene families like *Hsp70* [55]. In the wild, inversions covariate with life-history and fitness-related traits [9]. Until now, however, attempts to reproduce observed spatiotemporal patterns of inversions and their phenotypic associations under laboratory conditions have been largely unsuccessful [56, 57].

Many of the above and other findings would not have occurred without the previous development of the *cherry*-*curled* (*ch-cu*) recessive marker- [16] and the *Varicose*/*Bare* (*Va*/*Ba*) balancer-strains [58]. Motivation to use *D. subobscura* as a model to continue research on central issues of evolutionary biology is, however, presently hindered by the lack of a reference genome for the species. Recently, one step to narrow this gap was taken with the publication of a short-read second-generation Illumina-based genome of *D. guanche* [59]. In this paper, we took an additional step using flow-cytometry and long-read third-generation single-molecule real-time (SMRT) PacBio technology, together with the available wealth of genetic marker and synteny data, to assemble and annotate a high-quality nuclear and complete mitochondrial genome for *D. subobscura*, from our laboratory stock of the *ch-cu* strain. Long-read based assemblies are advantageous over short-read based ones because they are better at traversing across common repetitive structures, which results in more contiguous and complete assemblies. Our goals were two-fold. First, to provide a preliminary account of main features of the newly assembled genome and, second, to perform a comparative synteny analysis with *D. guanche* to trace the evolutionary history of fixed chromosomal rearrangement in the *subobscura* subgroup. Until now, this latter issue has been approached using wholly cytological methods [14, 25, 60] which are coarse-grained compared to the single-nucleotide resolution furnished by comparative genomics.

Knowing the sequence identity of synteny breakpoints can help determine both the evolutionary polarity of chromosomal rearrangement states by comparison with an outgroup, and the mechanism of rearrangement formation through assessment of remains of its molecular footprints. *Drosophila* inversions are commonly thought to originate by one of two major mechanisms, namely ectopic recombination, and chromosomal breakage and subsequent repair (reviewed in [61]). The first mechanism predicts occurrence of duplications on the flanks of the inverted segment in both the ancestral and the derived arrangement states, whereas the second predicts absence of duplications or their presence only in the derived state. Knowing how an inversion originated can shed light on why it evolved [62]. Inversions can have direct, indirect, or both types of fitness effects. New inversions can themselves be direct targets of selection because of functional disruption by the breakpoints. The main importance of inversions, however, might stem indirectly from the fact that they suppress recombination in heterokaryotypes. Through their linkage generation effects, inversions can contribute to keep sets of adapted alleles together in the face of gene flow [63–65].

## Results and discussion

### Size estimation and de novo long-read assembly of the *D. subobscura* genome

The genome size of the inbred *ch-cu* line was estimated using *k*-mer counting and flow cytometry methods. By the first method, GenomeScope (http://qb.cshl.edu/genomescope/ [66]) analysis of 21-mer frequencies obtained by Jellyfish (Ver. 2.2.4. [67]) using 20 million Illumina short (300 bp) reads [55] resulted in a genome size of 136.943 Mb. By the second, flow cytometry of PI-stained female brain cell nuclei using a 328.0 Mb genome from *D. virilis* [68] as internal standard resulted in a genome size of 148.069 Mb (0.151 pg ± 0.001; for the mean plus/minus one standard deviation across five replicates; see Methods). This latest measure conforms to previous flow cytometry-based estimates of the *D. subobscura* genome size (146.7 Mb [69, 70]); rounded to 150 Mb, it was the value set as genome size for the Canu assembler.

The PacBio 7 SMRT cells sequencing of the *ch-cu* genome generated a raw output of 1,252,701 subreads, hereon referred to as reads, with mean and longest read lengths of 8003 bp and 52,567 bp, respectively (Additional file 1: Table S1). These sequences totaled 10,025,366,103 bp, or a ~ 67-fold estimated genome coverage. The average yield per SMRT cell (~ 1,4 Gb) was on the upper bound of the manufacturer range for the typical SMRT cell (0.75–1.25 Gb [71]), which highlights the suitability of the used high-quality genomic DNA isolation protocol. Canu correction and trimming of the PacBio data retained 1,060,943 reads of 6103 bp average read length, or the equivalent to a 43-fold genome coverage for the assembly, well within Canu’s default sensitivity range (30-fold to 60-fold) (Additional file 1: Table S1). Of the 327 Canu-generated contigs, 115 (totaling 6624 Mb) showed evidence of foreign sequences. All the contigs in this subset were solely of bacterial origin, each being exclusively either from *Acetobacter* or from *Providencia*, which are genera known to be part of the *Drosophila* microbiome [72]. After removing these contigs, the primary Canu assembly consisted of 212 contigs spanning 129.183 Mb, with an N50 of 3.129 Mb and a maximum contig length of 15.083 Mb (Additional file 1: Table S1).

A first round of quality control and scaffolding of the Canu contigs carried out combining recursively i) automated BLAT and BLASTN searches against the *D. melanogaster* and *D. pseudoobscura* genomes, and ii) evaluation of consistency with published data on the chromosomal position of 621 cytological (604) and genetic linkage (17) markers (Additional file 2: Table S2; see Methods) did not detect any misassembling. Scaffolding of the Canu contigs using SSPACE-LongRead (Ver. 1–1. [73]) resulted in 157 scaffolds. Submission of these scaffolds to a second round of quality control and scaffolding as in step one resulted in 186 validated scaffolds with a total length of 129,237 Mb (Additional file 1: Table S1). Half of the assembly was in 7 scaffolds longer than 5.954 Mb, while an additional 45% was in 44 scaffolds longer than 313 Kb. The GC content of the assembly was 45.0%, similar to that found for the close relative *D. pseudoobscura* (45.3%; r3.04 assembly [74]). Based on the available cytogenetic and genetic linkage marker data, it was possible to assign confidently genomic coordinates to 96.6% of the assembled sequence (63 scaffolds spanning 124,862 Mb, with half of it in 6 scaffolds longer than 8.237 Mb). On average, there were 10 markers per scaffold. A total of 38 scaffolds, representing 91.4% of the positioned sequence, were placed using ≥2 markers. The remaining 25, relatively shorter scaffolds with only 1 marker (10; average length 0.656 Mb) or 0 markers (15; 0.363 Mb) were placed confidently aided by synteny-based inferences of orthology with the close relative *D. guanche* and/or with *D. melanogaster*. Detailed information about the markers used for the anchoring, ordering and orientation of the scaffolds is provided in Additional file 2: Table S2.

**Fig. 1:**
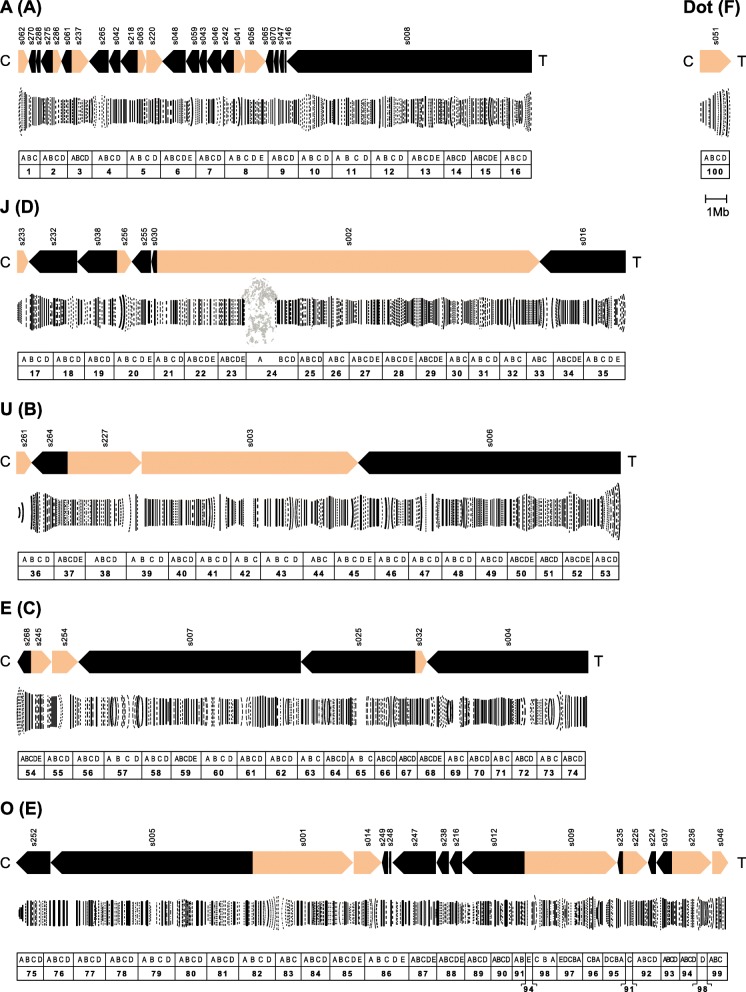
De novo assembly of a *D. subobscura* genome from long-read PacBio sequencing data. The six chromosomes are referred to by their corresponding letter (i.e., A, J, U, E, O and dot) and Muller element (i.e., A, D, B, C, E and F, respectively; in parentheses) designations. Chromosomes are shown oriented from centromere (C) to telomere (T). Each chromosome panel includes (top) a scheme of the reconstructed pseudochromosome and their component forward (sepia) and reverse (black) scaffolds with labels (e.g., s062) on them; (center) a drawing of the Kunze-Mühl and Müller [12] reference standard karyotype, modified to take into account that the *ch-cu* strain used for genome sequencing is structurally O_3 + 4_ (or O_ms + 4_; see the results and discussion section) and (bottom) a ruler indicating the sections (from 1 to 100) and subsections (each from A to E) of the Kunze-Mühl and Müller [12] map. A 1 Mb-scale bar is shown below the dot

The final assembly resulted in six chromosome-sized pseudomolecules or pseudochromosomes, one for each of the six chromosomes of the haploid *ch-cu* female chromosome set (Table 1 and Additional file 1: Table S1; Fig. 1). The pseudochromosome dot consisted of a single contiguous sequence 1.376 Mb long; the A incorporated 24 scaffolds spanning 22.858 Mb (the largest being 11.265 Mb long; coordinates assigned based on 123 markers); the J, eight scaffolds spanning 23.583 Mb (15.120 Mb; 45); the U, five scaffolds spanning 25.800 Mb (11.275 Mb; 21); the E, seven scaffolds spanning 20.819 Mb (8.237 Mb; 293); and the O, 18 scaffolds with combined size of 30.426 Mb (8.841 Mb; 140). The number of scaffolds is greater for chromosome A than for the autosomes, probably because we sequenced genomic DNA from a pool of 50:50 males and females, such that the A would be expected to have three-quarters the sequence coverage of the autosomes. The lengths of the pseudochromosomes show nearly perfect correlation with the linear lengths of the corresponding polytene chromosomes measured from the Kunze-Mühl and Müller [12] reference map (Pearson’s *r* = 0.99; *P* < 10^− 4^). While the rest of the assembly not included in the pseudochromosomes (3.4%; 4.375 Mb in 123 scaffolds) could not be assigned precise genomic coordinates owing to non-availability of reliable positioning information, for most of it (81.2%; 3.551 Mb in 90 scaffolds) it was possible to at least anchor it to chromosomes (including the rDNA chromosome) using similarity search tools (Additional file 1: Table S1). Only 0.6% (0.824 Mb in 33 scaffolds) of the assembly remained completely unplaced. This included cases where either there was no marker/synteny data available, or the placement of the corresponding BLAT/BLASTN hits in the reference species is unknown.

**Table 1:** Overview of *D. subobscura* nuclear pseudochromosome and mitochondrial reference genome assembly (N50: length of the contig for which 50% of the total assembly length is contained in scaffolds of that size or larger; L50: ranking order of the scaffold that defines the N50 length; lengths are in bp)

Component	Length	No. of scaffolds	Largest scaffold	L50	N50	Gene models	% repetitive
Nuclear	124,861,819	63	15,119,984	6	8,236,782	13,181	11.7%
Dot	1,375,632	1	1,375,632	1	1,375,632	91	28.9%
A	22,857,882	24	11,265,230	2	1,077,607	2322	14.6%
J	23,583,473	8	15,119,984	1	15,119,984	2452	11.1%
U	25,800,175	5	11,274,558	3	9,313,524	2496	10.3%
E	20,818,511	7	8,236,782	2	5,954,457	2591	11.3%
O	30,426,146	18	9,011,354	3	4,063,992	3229	10.6%
Mitogenome	15,764	1	15,764	1	15,764	37

### Ab initio gene prediction and functional annotation

The complete *ch-cu* assembly was predicted to contain 13,939 protein-coding genes, nearly the same number as in the current release of the *D. melanogaster* genome (13,931; r6.18 assembly [75]). Of them, 13,317 (95.5%) were successfully annotated by the MAKER annotation pipeline, which corresponds to a gene density of one gene every 9.70 kb of the genome assembly. The average gene length was 3.502 kb. All genes combined span 46.635 Mb of coding sequence, with a GC content of 55.6%. The average number of exons and introns per gene was 4.6 and 3.6, with average (median) exon and intron lengths of 379 (213) bp and 529 (66) bp, respectively. A total of 87.2% of the genes were multi-exonic.

Of the 13,317 annotated protein-coding genes, 13,181 (99.0%) are placed in the six pseudochromosomes that were assigned genomic coordinates (Table 1). The numbers of annotated genes per pseudochromosome (dot: 91; A: 2322; J: 2452; U: 2496; E: 2591; and O: 3229) depart from the expected from pseudochromose length (G = 113.61; d.f. = 5, *P* < 10^− 6^), with E and U showing, respectively, the greatest excess (393 genes) and deficiency (228), in line with previous findings in *D. melanogaster* [76]. With respect to the small subset of genes that could no be assigned precise genomic coordinates (139), most of them (89) were anchored to chromosomes. In addition, 3090 non-coding genes were annotated, including 1191 and 1899 short and long non-coding RNA genes, respectively. Of note, the 5S rDNA gene family was found to consist of > 160 copies of the 5S rDNA repeat unit, tandemly arranged in one cluster located on the distal end of segment II of autosome O, in agreement with early in situ hybridization results [77]. Also, we identified > 80 copies of the 18S–28S rDNA repeat unit distributed over the 19 rDNA annotated scaffolds. With respect to the relatively more rapidly evolving lncRNA genes, BLASTing with FlyBase lncRNA (Dmel_Release_6) detected 1898 out of the 2965 lncRNA annotated genes, with a strong bias towards the longer ones (10.2 kb vs. 1.2 kb, for the average lengths of detected vs. undetected lncRNAs, respectively).

The high-quality of the genome assembly and annotation is further buttressed on three validation metrics. Firstly, the overall size of the assembly (129.237 Mb) closely matches the estimated size of the genome using the *k*-mer counting (94.4% of 136.943 Mb) and flow-cytometry (87.3% of 148.069 Mb) methods. Secondly, both the low values of the average and median of the MAKER-defined AED scores (0.127 and 0.070, respectively), and the fact that nearly all genes attained AED scores lower than 0.5 (AED_50_ = 97.9%) are indicative of a good agreement between the annotations and their evidence. And thirdly, BUSCO analysis using the 2799 25-dipterans orthologous gene set resulted in 96.5% (2671) single complete genes, 0.5% (14) duplicated complete genes, and 3.0% (84) fragmented. Only 1.1% (30) of the BUSCO genes were missing, indicating that the assembly is nearly complete.

### Phylogenetic placement of the *D. subobscura* genome and age of the *subobscura* subgroup

To further assess the quality of the obtained genome, we subjected it to a phylogenetic analysis together with closely related species with known relationships. We took advantage of the carefully curated 12 *Drosophila* multiple sequence alignment (MSA) data set used by Obbard et al. [78] (see also [75]). The MSA consists of 67,008 characters from 50 concatenated nuclear protein-coding loci selected for (i) having only 1:1 orthologs, (ii) including an exon longer than 700 bp, and (iii) not showing unusually high codon usage bias. To this MSA, we added the corresponding reciprocal-BLAST-identified orthologs from *D. subobscura* and *D. guanche* using MAFFT (Ver7; http://mafft.cbrc.jp/alignment/software/), and then identified the best-fit model of sequence evolution (GTR + G + I; with α = 0.53, and I = 0.27) for maximum-likelihood (ML) tree estimation using MEGA7 [79]. The resulting tree topology (Additional file 3: Figure S1) is consistent with the known phylogeny of the species. Using this topology, and the RelTime-ML method [80] with the mutation rate-based estimates found by Obbard et al. [78] to perform best as calibration dates, the age of the *subobscura* species subgroup was found to be 1.72 ± 0.51 Mya (Additional file 3: Figure S1). This estimate is at the lower bound of published dates for this divergence, which were all based on one or few available markers (ranging from 1.8 to 8.8Mya, median 2.75Mya [27, 81–84]).

### Mitochondrial genome identification and annotation

**Fig. 2:**
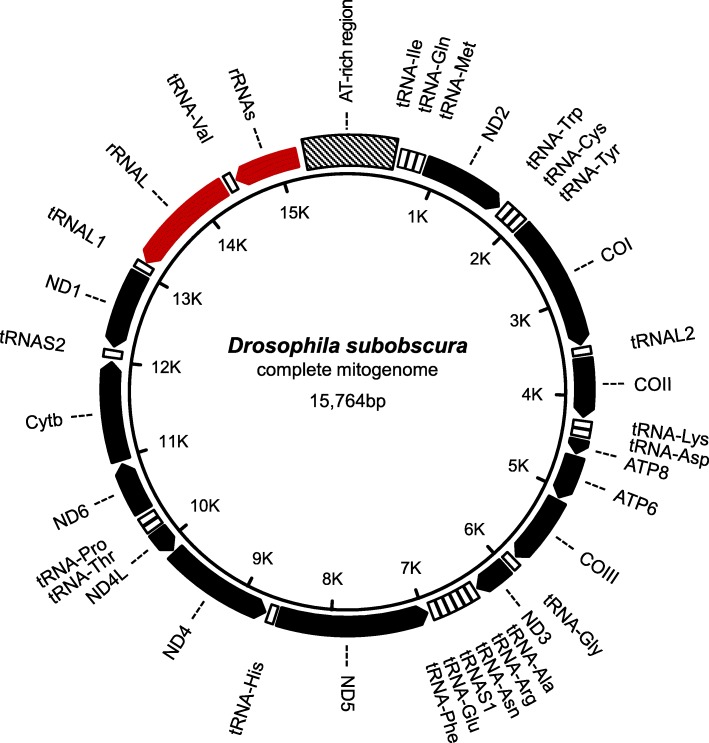
*D. subobscura* mitogenome content and organization. Shown are protein coding genes (black), rRNA genes (red), tRNA genes (white), and the AT-rich (control) region (crosshatched). Arrowheads indicate gene direction

BLASTN searches against the *ch-cu* assembly found that Canu’s tig00002375 contained a complete copy of the *D. subobscura* mitogenome. The mitogenome is 15,764 bp long, and shows the gene number, order and orientation of the typical insect (Table 1 and Additional file 4: Table S3; Fig. 2 [85]), including 13 PCGs (*ND1–6*, *COI-III*, *ND4L*, *Cytb*, *ATP6*, *ATP8*), 2 ribosomal RNAs (*lrRNA* and *srRNA*), 22 tRNAs, and an AT-rich region (control region). The control region is 944 bp long, and is placed between genes rRNAS and tRNAI. The nucleotide composition is biased towards A + T (78.3%), the bias being greatest in the control region (93.0%). The plus strand codes for 23 genes (9 PCGs and 14 tRNAs) and the control region, while the minus strand codes for the remaining 14 genes (4 PCGs, 8 tRNAs and 2 rRNA genes). All PCGs start with the typical ATN codons, except *COI* that starts with a TCG codon, and terminates with the TAA/TAG codons, except *COII* and *ND5* that end with the incomplete T stop codon. Furthermore, there is a pattern of nucleotide overlap for five pairs of genes: *tRNAW*–*tRNAC* (7 nt), *ATP8*–*ATP6* (5 nt), *ND4L*–*ND4* (3 nt), *tRNAL1*–*rRNAL* (41 nt), and *rRNAL*–*tRNAV* (13 nt). The size of the mitogenome is within the range found in *Drosophila*, from 15,641 bp (*D. incompta* [86]) to 19,524 bp (*D. melanogaster*; Unpublished, GenBank accession number: NC_024511.2). Absence of the diagnostic HaeIII restriction site (GG/CC) in the *ND5* gene indicates that, of the two major mitotypes segregating in *D. subobscura* populations [19], the one obtained in this study is derived from mitotype II.

As a quality check, the obtained mitogenome was subjected to a phylogenetic analysis with available mitogenomes from the same 13 *Drosophila* as above. As the mitogenomic sequence from *D. guanche* [59] was found containing multiple unusual features as to size (20.7 kb) and number of duplications and rearrangements, this analysis was focused only on the PCG regions. We performed multiple sequence alignment (MSA) separately for each of the 13 PCGs using MAFFT (version 7; http://mafft.cbrc.jp/alignment/software/ [87]) followed by evaluation of the 13 MSAs using Gblocks [88] and visual inspection. Then, we merged the MSAs into a single, 11,244 characters long MSA, identified the best-fit maximum-likelihood evolutionary model of the concatenated MSA (GTR + G + I; with α = 0.29, and I = 0.34), and used this model to find the maximum-likelihood tree using MEGA7 [79]. The resulting mitogenomic tree topology was concordant with the known phylogeny of the species (Additional file 3: Figure S1).

### Genomic distribution of repetitive DNAs

A 14.3% of the *ch-cu* genome was annotated as repetitive (Additional file 5: Table S4). This density of repetitive sequence is low compared to estimates from other *obscura* group species, including the close relative *D. guanche* (30% [59]), and the more distantly related *D. pseudoobscura* (23.9%; release R3.04) and *D. persimilis* (39.0%; release R1.3). That *D. subobscura* has a relatively compact, less repetitive genome is further supported by available measures of genome size obtained using flow cytometry from brain cell nuclei: its genome is nearly 30 Mb smaller than that of *D. guanche* (167.230 Mb), and the smallest of the ten *obscura* group species values stored in the animal genome size database [70, 89].

Repetitive DNAs were analyzed by classifying them into five categories: long terminal repeat (LTR) and non-LTR retrotransposons, DNA transposons, satellites (including *sat290* and *SGM-sat*), and simple repeats or microsatellites. The extrinsic null of no deviation in repeat number content from the expected from relative chromosomal length was tested using G-tests among all six chromosomes, between A and the four large autosomes, and among the four large autosomes. Overall, there were significant differences in proportion of repetitive sequence among the six chromosomes, whether repeats were considered together or separately by category (all G-tests: *P* < 10^− 6^). The dot showed the largest aggregated excess (2.5-fold; 2.7% of total repeat number content), because it showed 2.6 (3.0%), 4.1 (4.8%) and 4.9-fold (5.7%) more non-LTRs, DNA transposons and satellites than expected from its length, whereas A was the only chromosome that showed a consistent excess of repetitive sequence across all five repeat categories, particularly microsatellites (1.5-fold; 26.9%). Comparatively, the four large autosomes showed a dearth of repetitive DNA. When the dot and the A were excluded from the analysis the magnitude of the deviations in amount of repetitive sequence dropped markedly [with the single exception that the E chromosome shows a 1.3-fold (27.7%) excess of non-LTRs], and no definite pattern emerged.

**Fig. 3:**
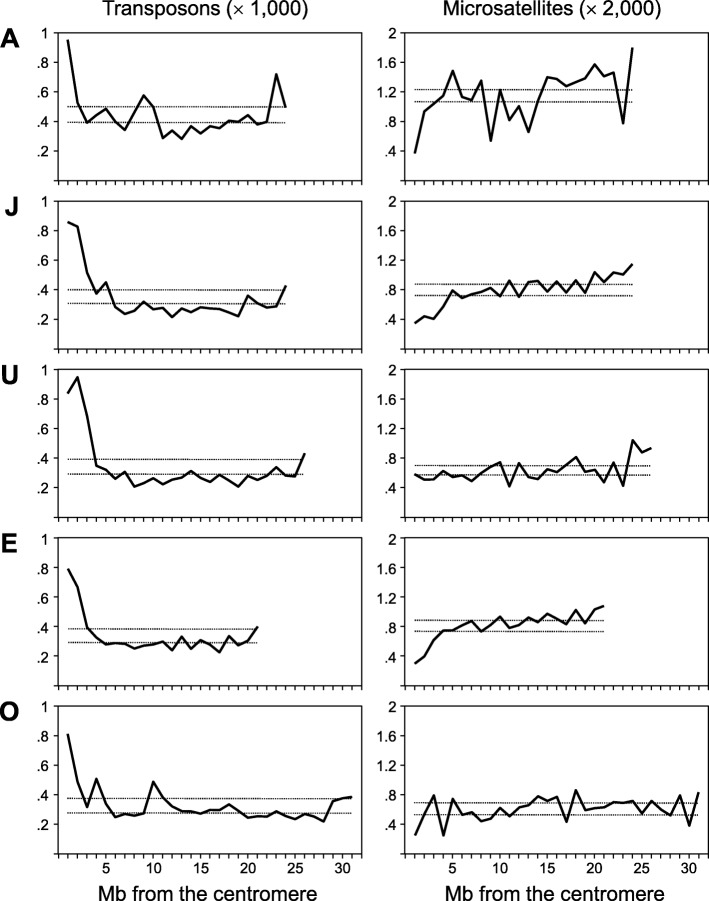
Spatial distribution of two major categories of repetitive DNA along the five large acrocentric pseudochromosomes of the *D. subobscura* assembly. Dotted lines represent 95% confidence intervals around the expected average if repeats were distributed at random

The distribution of repetitive DNA densities along chromosomes is shown in Fig. 3. For simplicity, non-LTR and LTR retrotransposons, DNA transposons and satellites were aggregated into a single class separately from microsatellites. The two classes differ qualitatively in their patterns of chromosomal distribution. Transposable elements and satellites appeared concentrated in the pericentromeric and (less so) peritelomeric regions. This was so particularly for DNA transposons and satellites, and the pattern became most apparent for the J and U chromosomes. In addition, there were large megabase-scale regions with high density of DNA transposons and satellites in the A and O chromosomes. Interestingly, the distal-most peak of repetitive sequence in chromosome A, in fact consists of telomeric sequence that was repositioned to that location by inversion A_6_ (see below). Microsatellites deviate markedly from this pattern, showing nearly monotonic trends to increasing density towards the telomeres, that became statistically significant for the J, U and E chromosomes (simple linear regressions: *r*^*2*^ = 0.73, *P* < 10^− 5^; *r*^*2*^ = 0.25, *P* = 0.009; and *r*^*2*^ = 0.65, *P* < 10^− 4^, respectively). Understanding the significance of these differences warrants further in-depth analyses.

Satellites *Sat290* and *SGM-sat* have gathered special interest. *Sat290* is a 290 bp repeat satellite [90]. Early in situ hybridization studies in the three members of the *subobscura* subgroup concluded that *sat290* was absent in *D. madeirensis* and *D. subobscura*, and that in *D. guanche* the repeat comprised a major satDNA class distributed in centromeric regions [90]. *SGM*-*sat* would be derived from the MITE-like *SGM*-*IS* transposable element that was already present in the last common ancestor of the *obscura* group [91]. The repeat underwent a species-specific expansion in *D. guanche*, which gave rise to another major satDNA class in this species. Some of these findings were reassessed by a recent study of the *D. guanche* genome combining Illumina short-read whole genome sequencing and dual-color fluorescence in situ hybridization [59]. The study found *sat290* and *SGM-sat* to comprise the first and second most abundant satDNAs of *D. guanche*, respectively, adding up to ~ 30% of the species’ genome. In addition, *SGM*-*sat*s were found to be concentrated in the centromeres, but in more peripheral positions relative to the chromosome ends than *sat290*s. In contrast with this picture, our initial characterization of these repeats in the *ch-cu* assembly showed that *sat290* is present in *D. subobscura*, and in non negligible numbers (637), of which nearly one half are dispersed throughout the euchromatin. *SGM* showed a similar pattern, but conversely to the situation in *D. guanche*, in *D. subobscura SGM* sequences are 8-fold more abundant than *sat290*s (Additional file 5: Table S4).

Overall, our preliminary screen of the genomic distribution of repetitive DNAs did not find evidence of an association between repeat density and numbers of segregating chromosomal rearrangements. For example, the J and E chromosomes, which are about the same size show comparable percentages and distributional patterns of repetitive sequence (~ 11.0%; Fig. 3), in spite that the former exhibit 4-fold lower number of polymorphic inversions than the latter (5 vs. 22, respectively [9]).

### Orthologous group assignment and variation in gene family size

OrthoMCL clustered the 152,068 PCGs in the *Drosophila* pan-genome dataset into 23,394 orthologous groups, of which 8390 (35.9%) formed the core set shared by all 14 species (Additional file 6: Figure S2). Of this core set, 6293 were single-copy gene families. *D. subobscura* contained 10,483 orthologous groups, including 904 (8,6%; 965 genes) lineage-specific, of which 867 were single-copy orphans. These numbers and categories of orthologous groups are similar to those obtained for its close relative *D. guanche* in a previous comparison of the same 13 *Drosophila*, excluding *D. subobscura* (10,417 orthologous groups, including 838 species-specific, of which 828 were orphans [59]). Also, the number of orphan genes in *D. subobscura* is within the range of those estimated for the other 13 *Drosophila*, which varies from 294 in *D. erecta* to 2341 in *D. persimilis* (Additional file 6: Figure S2).

CAFE analysis (see the Methods section for details) carried out without taking into account variation in genome quality across genomes indicates that the best description of the data is provided by the five λ model, which distinguishes average fast- (λ_F_), medium- (λ_M_) and slow-evolving (λ_S_) branches, in addition to allowing the terminal branches leading to *D. subobscura* and *D. guanche* to have their own rates (λ_Ds_ and λ_Dg_; Additional file 7: Table S5). According to this model, the rate of gene family size evolution in these two lineages would be of the same order of magnitude as the average fast rate (λ_Ds_ = 0.0257 and λ_Dg_ = 0.0191, vs. λ_F_ = 0.0216). Adding a global error term (ε) improves the model fit significantly (−2ΔL = 191,98; *p* < 1 × e^− 6^; 1 *d.f.*), which indicates an effect of variation in quality across genomes. The effect, measured as the ratio (λ-λ_ε_)/λ [92], is lowest for slow-evolving lineages (24%) and *D. subobscura* (24%), and largest for fast-evolving lineages (43%) and *D. guanche* (41%). The best score of *D. subobscura* compared to *D. guanche* according to this criterion may be a reflection of a greater contiguity of the assembly provided by the PacBio long-read sequencing used in the first case, compared to the Illumina short-read sequencing used in the second.

The CAFE five λ model with global error term indicates that, of the 9155 gene families inferred to have been present in the *Drosophila* most recent common ancestor, 567 have increased and 636 decreased in size in the terminal branch leading to *D. subobscura* (Additional file 8: Figure S3). Of them, 62 show significant expansions (43; 272 genes) or contractions (19; 121 genes) relative to the genome-wide average (*P* < 0.01; Additional file 9: Figure S4). Functional enrichment analysis of the rapidly evolving families showed the expanding and contracting families to be significantly enriched for 52 and 77 GO terms, respectively (Additional file 10: Table S6 and Additional file 11: Table S7). Most-encompassing GO terms associated with the families that have expanded include, among others, ‘*thermosensory behavior*’ (GO:0040040) (Additional files 12, 13 and 14: Figures S5-S7), and those associated with the families that have contracted include ‘*sensory perception of sound*’ (GO:0007605) and ‘*response to red light*’ (GO:0010114) (Additional files 15, 16 and 17: Figures S8-S10). These terms appear particularly noteworthy considering the continuing role of *D. subobscura* as a model for research on insect thermal biology, and that, as previous research has shown, the species may be unique within the *obscura* group in not producing courtship auditory cues by wing vibration [8], and being unable to mate in the dark [6, 7, 93]. We hope that these results will stimulate future research on the role that those gene families play in the functional biology of *D. subobscura*.

### Evolutionary history of chromosomal rearrangement in the *subobscura* subgroup

Comparative synteny mapping of the genome of *D. subobscura* with those of three increasingly distant relatives, namely *D. guanche*, *D. pseudoobscura* and *D. melanogaster* using SyMAP showed the amount of genome rearrangement to scale up with evolutionary distance. Aggregated across the five Muller elements, *D. subobscura* synteny with each of the aforementioned species is fragmented into an increasingly larger number of increasingly smaller blocks: 31 blocks of 3.952 Mb average size (13 inverted), 333 of 0.345 Mb (164), and 540 of 0.220 Mb (264), respectively (Additional file 18: Table S8 and Additional file 19: Table S9). Chromosome A shows the greatest degree of synteny fragmentation in all three pairwise species comparisons (12, 90, and 125 vs. 5, 61, and 104 blocks, for A vs. the average autosome), in agreement with reported higher rates of rearrangement evolution for this Muller element compared to the autosomes [65, 94].

**Fig. 4:**
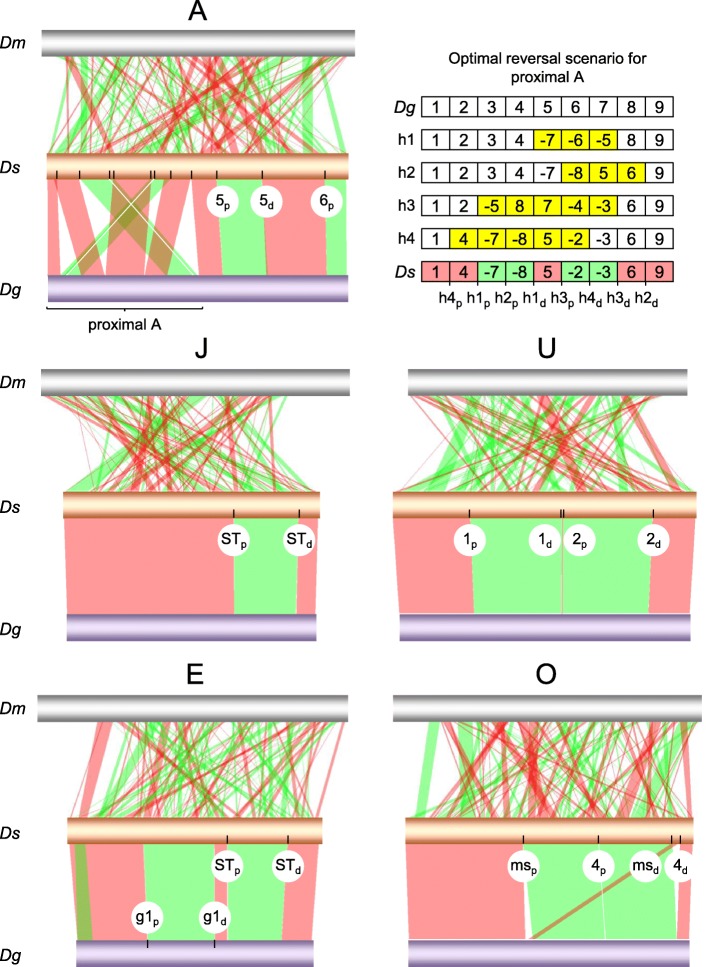
SyMAP comparative chromosome synteny analysis between *D. subobscura* (*Ds*; central gold horizontal bars) and each of *D. melanogaster* (*Dm*; upper grey) and *D. guanche* (*Dg*; bottom purple). Bands connecting homologous chromosomes denote noninverted (pink) and inverted (green) synteny blocks. Labeled ticks on chromosomes indicate proximal (p) and distal (d) inversion breakpoints. Labels for breakpoints in the proximal region of the A chromosome are provided in the upper right panel of the figure (h1_p_ to h4_d_), along with the optimal reversal scenario for the transition between the standard sequence of *D. subobscura* and the arrangement of *D. guanche* in this region inferred using the GRIMM algorithm. The eight synteny blocks of that transition are designated by positive (noninverted) and negative (inverted) numbers, and the corresponding four intermediate hypothetical inversions (yellow) by letter “h” subscripted 1–4. Cytological map positions and pseudochromosome coordinates of inversions breakpoints are given in Additional file 21: Table S10

Identified synteny blocks between the *D. subobscura* and *D. guanche* genomes have associated 28 breakpoints (11, 2, 4, 5 and 6, for the A, J, U, E and O chromosomes, respectively), of which 25 could be ascribed to 13 large-megabase scale paracentric inversions as shown in Fig. 4. To simplify matters, in that figure and henceforth, we used subindex “a” to denote ancestral arrangements of the species subgroup (except for U_1 + 2_, because it is shared by the three species), “g” for inversions fixed in the lineage of *D. guanche*, “ms” for inversions fixed in the most recent common ancestor of *D. madeirensis* and *D. subobscura*, and “h” for hypothetical rearrangement steps invoked to interconvert alternative gene arrangements. Of the 13 rearrangement differences, 6 occurred in chromosome A, including 4 overlapping inversions in its proximal half (A_h1_-A_h4_), and 2 single inversions in its distal half (A_5_ and A_6_); and 1, 2, 2 and 2 inversions in autosomes J (J_ST_), U (U_1_ and U_2_), E (E_g1_ and E_ST_) and O (O_ms_ and O_4_), respectively. With respect to the proximal half of chromosome A, 4 overlapping inversions is the minimum number of reversals required to interconvert the gene arrangements of the two species in that region [60]. Figure 4 (upper right) depicts one of those hypothetical paths (in fact, the only one consistent with Ah being the newest; see below) inferred using the algorithm implemented in GRIMM (http://grimm.ucsd.edu/GRIMM/ [95]), taking into account the ordering and orientation of the observed 9 syntenic blocks. Overall these results corroborate previous cytological ideas as to the number of paracentric inversion differences between the two species [14, 60].

Of those 13 rearrangement differences, nine are thought to be fixed between the two species, including A_h1_-A_h3_, A_5_ and A_6_, J_ST_, E_g1_ and E_ST_, and O_ms_; three are thought to be fixed in *D. guanche* and polymorphic in *D. subobscura*, including A_h4_ (assumed to be the same as *D. subobscura*’s A_1_), U_1_ and U_2_; and one, namely O_4_, it is found only as polymorphic in *D. subobscura* [14] (here, it may be helpful to recall that the *ch-cu* homokaryotypic strain used to represent *D. subobscura* is *standard* for all chromosomes except chromosome O, for which it is O_3 + 4_; see below). For none of these 13 inversions, except O_4_ [34], the nucleotide sequences of their breakpoints have been molecularly characterized. Yet this knowledge could allow testing current cytology-based ideas about the identities and evolutionary polarities of the rearrangement states, as well as ascertaining their originating mechanisms through assessment of remains of their molecular footprints.

To further validate the high quality of the newly obtained *D. subobscura* genome, we applied it to determining the unknown breakpoint sequences of the aforelisted 12 inversions as follows (Additional file 20: Figure S11). We defined synteny breakpoint as the nucleotide interval between contiguous SyMAP synteny blocks. Suppose two orthologous gene arrangements A|BC|D and A|CB|D in taxa 1 and 2, respectively, where the second arrangement is identical to the first one, but for the inverted sequence CB, with the vertical lines denoting the inversion breakpoints. If e.g. region A|B, spanning the proximal breakpoint in taxon 1 plus 5 kb towards the inside of each of its two flanking synteny blocks is BLASTed against the genome of taxon 2, there should produce two hits, one in locus A, and the second one in locus B. In addition, each hit should carry associated an alignment overhang due to lack of homology between B and C, and between A and D, respectively; and the coordinates of the hits should match the SyMAP coordinates for the breakpoints spanning the inversion. Furthermore, the results from breakpoints of the same inversion must be reciprocally consistent, regardless the taxon used as query.

By the above described approach, we were able to isolate and characterize the putative breakpoint sequences of all the 12 targeted inversions. The results challenge previous cytology-based assumptions about the identity and evolutionary polarity for some of the rearrangement states. Additional file 21: Table S10 and Fig. 4 summarize the main results. The proximal half of chromosome A provides an all-embracing example. In this region, the structural transition between the two genomes requires minimally four inversions (A_h1_-A_h4_; Fig. 4). Cytological evidence for shared breakpoints supporting that A_h4_ is the same inversion as A_1_, led to postulate that A_h1_-A_h3_ were fixed in the lineage of *D. guanche* [14, 25]. Recently, the proximal and distal breakpoints of inversion A_1_ segregating in *D. subobscura* were assessed by a mixed approach combining cytological and molecular methods [96]. Although the attempt was unsuccessful, it managed to narrow them down to within a few kilobases distal to the markers *cm* (CG3035) and *dod* (CG17051), respectively. The coordinates of those markers in the newly obtained *ch-cu*, i.e., A_ST_, genome (chrA:1,206,180 bp and chrA:8,875,126 bp, respectively) lie more than half a megabase proximal to their corresponding nearest breakpoint of the A_h4_ inversion separating the *ch-cu* strain from *D. guanche* (chrA:692,605 bp and chrA:8,198,726 bp; Additional file 21: Table S10). This finding indicates that the previously supposed-to-be same inversion shared by the two species, i.e., A_h4_ equal to A_1_, in fact represents two different inversions that originated separately.

Comparative analysis of gene arrangement of the regions around the breakpoints of A_h4_ in *D. subobscura* and *D. guanche* with those in the outgroup *D. melanogaster* indicates that *D. guanche* shows the ancestral arrangement state (CG2076, CG2081, CG18085, |, CG15203, CG1537, CG1545, and CG32677, CG43347, CG1628, |, CG15302, CG32683, CG2096; for the proximal and distal breakpoints, respectively, in both *D. guanche* and *D. melanogaster*; with the vertical lines denoting the inversion breakpoints; Additional file 21: Table S10), whereas *D. subobscura* shows the derived state (i.e., CG2076, CG2081, CG18085, |, CG1628, CG43347, CG32677, and CG1545, CG1537, CG15203, |, CG15302, CG32683, CG2096; Additional file 21: Table S10). In addition, no evidence was found for duplicated and/or repetitive sequences in the breakpoint regions from reciprocal BLAST searches, which supports that the inversion originated through a chromosomal breakage mechanism, either straight-breaks, or nearly straight-breaks, i.e., staggered-breaks whose resulting duplications are too short to leave long-lasting traces [61]. Be that as it may, no gene was found to have been directly disrupted by the inversion, suggesting that A_h4_ may have been favored indirectly because of its recombination suppression effects.

Apart from the example of A_h4_, it is worth pinpointing the cases of A_6_ and the pair U_1_ and U_2_. The first inversion seems a reversal of the telomeric end of chromosome A. Alternatively, it could be subtelomeric [60], and that the tip of the chromosome not affected by the inversion was not included in the assembly. In any case, the rearrangement produced the peritelomeric peak of transposable element repetitive content shown in Fig. 3. With respect to the pair U_1_ and U_2_, available cytological evidence could not distinguish between the distal breakpoint of U_1_ and the proximal breakpoint of U_2_, pointing to an instance of breakpoint reuse [9]. However, from the assembly the two breakpoints are clearly distinct, although they are only 31 kb distant from each other (Additional file 21: Table S10).

**Fig. 5:**
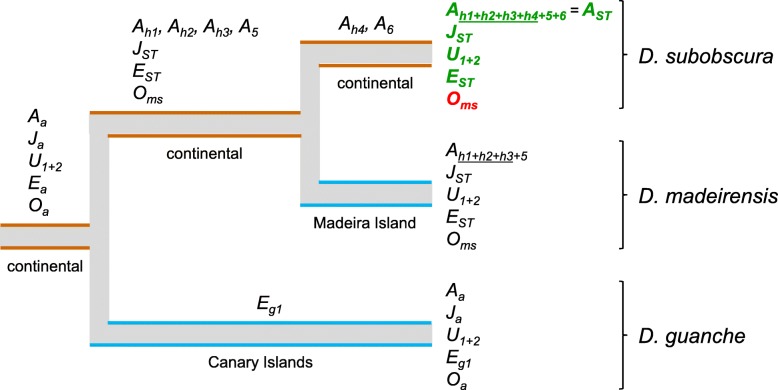
Reconstructed most parsimonious chromosomal rearrangement history of the *D. subobscura* species subgroup. Shown are the continental lineage (brown) leading to *D. subobscura*, and the two derived island lineages (blue) *D. guanche* and *D. madeirensis*. The inferred ancestral chromosomal arrangement configuration of the subgroup is shown at the root. Arrangements at the terminal nodes can be fixed (black), polymorphic (green) or extinct (red). Worth to mention is the case of O*ms*. This arrangement was previously known as O*g*, because, based on cytogenetic evidence, it was thought to have originated in the lineage of *D. guanche*. The breakpoint sequence synteny analysis conducted herein, however, indicates that the arrangement originated in the mainland before the split of *D. madeirensis*, where it became fixed, and *D. subobscura*, where superimposed on it originated separately inversions 4 and ST, and then became extinct. Accordingly, *D. subobscura* presently polymorphic O inversions 4 and ST may be more appropriately referred to O_ms + 4_ and O_ms + ST_, rather than O_3 + 4_ and O_ST_, because arrangement 3 is ancestral to ms

Figure 5 shows reconstructed most parsimonious evolutionary trajectories for all the 12 targeted inversions. Inclusion of *D. madeirensis* was because it is nearly homosequential with *D. subobscura*, and thought to be karyotypically monomorphic for inversions [15]; and also because, together with *D. guanche*, they are the small oceanic-island endemics of the species subgroup. Of the 12 inversions, all but one would have originated in the continental lineage leading to the presently inversion-rich *D. subobscura* (A_h1_ to A_h4_, A_5_, A_6_, U_1_, U_2_, J_ST_, E_ST_ and O_ms_), whereas only one became fixed in the inversion-poor island lineages (E_g1_ in *D. guanche*). Of note is the case of O_ms_, previously denoted O_*g*_ for it was thought to have originated in *D. guanche*. If it is considered that in *D. subobscura* O_ms_, rather than O_3_ as previously thought, is the immediate ancestor on which presently segregating ST and 4 arose, then it may be pertinent to rename O_ST_ and O_3 + 4_ to O_ms + ST_ and O_ms + 4_, respectively.

Between lineages, considering only rearrangement replacements, *D. subobscura* has evolved at a rate of 5.6 inversions/Myr (assuming 1.72 Myr to the common ancestor of the species subgroup; Additional file 3: Figure S1), which is over 10 times higher than that for the average island endemic (0.4 inversion/Myr; assuming 0.92 Myr to the split of *D. madeirensis* [27]). The difference is highly significant (*P* = 3.3 × 10^−5^, Poisson distribution). The lower rate of rearrangement accumulation in *D. guanche* and *D. madeirensis* compared to that in *D. subobscura* could be a reflection of a lower rate of rearrangement formation in small-sized island species. Another, not mutually exclusive possibility is that the difference could be related to that *D. guanche* and *D. madeirensis* remained localized to the small oceanic islands in which they arose, which have maintained relatively homogenous conditions [97], whereas, in comparison, *D. subobscura* is vastly distributed across multiple contrasting environments with high dispersion. This latter situation was shown to result in increased rates of structural evolution, when the evolutionary fate of inversions is driven by their effect in keeping sets of positively selected alleles together against maladaptive gene flow [64].

The role of the inversions recombination-suppression effect as one driver of genome structural evolution in the *subobscura* subgroup is further supported by the observed ratios of chromosomal divergence to polymorphism between the A sex chromosome and the autosomes in *D. subobscura*. In this lineage, compared to the average autosome the A chromosome shows 8 times larger inversion fixation rate (0.44 vs. 3.5 inversions/Myr, respectively; *P* = 0.006, Poisson distribution; so-called “faster-X” pattern [98]), while 1.8 fewer presently segregating inversions (14 vs. 8, respectively [9]). These conclusions remain qualitatively the same after accounting for chromosome length. The observation of contrasting ratios of polymorphism to divergence between the A sex chromosome and the autosomes agrees with expectations from positive selection models of inversion evolution as byproduct of their recombination-suppression effects in the face of gene flow, which explain this pattern as resulting from: (i) the higher efficiency of negative selection against locally recessive maladaptive alleles at A-linked genes, whereby A-linked inversions would be expected to capture higher-fitness genotypes with greater probability of fixation; and (ii) the higher likelihood that recessive deleterious alleles generate associative overdominance on autosomes, which would hinder autosomal inversions from fixation [65].

While we may have identified a signature of indirect inversion effects in driving the observed non-random patterns of genome structure evolution in the *subobscura* subgroup, that would not preclude the contribution of other mechanisms. Two would seem be particularly plausible and better suited to be assessed with the data on hand, including mutational biases in the formation of new inversions and direct inversion effects. Overall, however, no positive evidence for any of these two mechanisms could be obtained in the present study. With respect to the first, the observed accelerated rate of structural evolution of the A chromosome compared to the autosomes in *D. subobscura* could result from a bias in the formation of inversions arising from the comparatively higher repetitive content of the A chromosome (for example, if inversions tended to originate by ectopic recombination [99]). However, reciprocal BLASTN searches using the inversions breakpoints did not detect evidence for an enhanced repeat-based formation of A-linked relative to autosomal inversions. With respect to direct inversion effects, we provide a discussion of our findings in the context of related results below.

Figure 5 shows that, in all five inversion-rich chromosomes of *D. subobscura*, presently segregating *standard* structural variants arose in the mainland after the split of *D. guanche*. In addition to these finding, all the 12 inversions were inferred to have originated by chromosomal breakage. In 4 of the cases, the presence of duplicated sequences in opposite orientation on the flanks of the derived rearrangement provided clear-cut evidence of an origin by staggered breaks, including U_1_ (689 bp-long duplication), U_2_ (1007 bp), E_ST_ (513 bp) and O_ms_ (538 bp) (Additional file 21: Table S10). The remaining 8 cases could have originated through straight- or nearly straight-breaks. In no case evidence for gene disruptions at the breakpoints could be found, which does not support direct positive selection on the inversions as a major driver of genome structure evolution in the *subobscura* subgroup (see above). Overall, our results suggest that chromosomal breakage is the dominant originating mechanism for inversions in the subgenus *Sophophora*. This contrasts with the situation in the subgenus *Drosophila*, in which inversions appear to originate mainly via ectopic recombination. Although its causes remain to be understood, this difference supports that inversions can arise by alternative major mechanisms in different lineages [61].

## Conclusions

We presented the first high-quality, long read-based nuclear and complete mitochondrial genome for *D. subobscura*, and applied it to a synteny analysis of the evolution of genome structure in the *subobscura* species subgroup. We found the sequenced genome to exhibit a relatively compact size, compared to known values from the *obscura* group. *SGM-sat* and *sat290* represent the first and second most abundant satDNAs classes, conversely to the situation in the close relative *D. guanche*. *D. subobscura* exhibits the highest rate of accumulation of paracentric inversions of its subgroup. All identified inversions originated by chromosomal breakage, which adds to the evidence favoring this as the prevailing mechanism of inversion formation in the *Sophophora* subgenus of *Drosophila*. No evidence for direct gene disruption at the inversions breakpoints was found. This observation, together with the finding of contrasting ratios of inversion fixation to polymorphism between the A sex chromosome and the autosomes, overall suggests that the evolution of genome structure in the lineage leading to *D. subobscura* has been driven indirectly, through the inversions recombination-suppression effects in keeping sets of adaptive alleles together in the face of the high dispersion ability of the species. We have built a genome browser and a BLAST server (http://dsubobscura.serveftp.com/) to facilitate the further use of this resource.

## Methods

### *D. subobscura* karyotype and chromosome arrangement designation

*D. subobscura* has six pairs of chromosomes: five acrocentric and one dot. The five acrocentric chromosomes are symbolized by the alphabet vowels capitalized: A (the sex chromosome; Muller’s element A, homologous to X in *D. melanogaster*); I, commonly replaced by J (D, 3 L); U (B, 2 L); E (C, 2R); and O (E, 3R) [9, 11]. The species karyotype is divided into 100 numbered sections as follows: A (1–16), J (17–35), U (36–53), E (54–74), O (75–99) and *dot* (100). Each section is subdivided into 3–5 lettered subsections (from A to E [12]).

Gene arrangements are denoted by subscripts next to chromosome symbols (ST: *standard*; otherwise: alternative arrangements to ST). Overlapping inversions are denoted by underlines below number subscripts [100]. The O chromosome has been particularly amenable for study of structural variation, for it is the only chromosome for which a balanced lethal strain (namely, the *Varicose*/*Bare*, or abbreviated *Va*/*Ba* balancer stock [58]) is available. By convention, the O chromosome is divided into two segments, designated I (sections 91 to 99) and II (sections 75 to 90), which are located distal and proximal to the centromere, respectively. The structural variant of the O chromosome used in this study is designated O_3 + 4_, a rearrangement of segment I thought to have originated by superimposition of inversion 4 on the ancestral, and now extinct in *D. subobscura* gene order O_3_. It may be worth noting here, that the findings herein show that the immediate ancestral state to inversion 4 is not O_3_, but arrangement O_ms_, previously called O_g_ because it was thought to have originated in *D. guanche* (see the Results and Discussion section, and Fig. 5 legend).

### *D. subobscura* lines

We used one inbred line for de novo complete genome assembly using PacBio long-read data. The inbred line was obtained by 10 generations of full-sib mating of progeny of a single gravid female from our highly homozygous laboratory stock of the *ch-cu* marker strain. The *ch*-*cu* strain was established by Loukas et al. [16] from flies descended from the “*β*-*ch*-*cu*-stock” [101, 102]. Structurally, it is homokaryotypic for the ST arrangements in all chromosomes except in chromosome O, for which it is homokaryotypic for the O_3 + 4_ configuration. Crossing schemes and the methods for polytene chromosome staining and identification are described elsewhere [36]. The assayed inbred line was stored frozen at − 80 °C immediately upon obtention.

### Genome size estimation by flow cytometry

Genome size of adult *D. subobscura ch-cu* was quantified from five replicates of brain cell nuclei using propidium-iodide (PI) based flow cytometry [89]. By this method, the size of a target genome is estimated by comparing stain uptake of the target genome (PI–fluor _*target*_), with that of a standard genome of known size (PI–fluor_*standard*_). A *D. virilis* strain with known 328 Mb genome size [68] was used as the standard.

Nuclei were extracted from samples of 10–80 °C—frozen heads from four-days-old ice-immobilized females, each including 5 heads from *ch-cu* and 5 heads from the standard. Each sample was transferred to a glass/glass homogenizer (Kontes Dounce Tissue Grinder 7 ml), ground on ice-cold LB Galbraith buffer using the large clearance pestle (pestle A), and the homogenates filtered through nylon mesh (20 μm). The filtrates were stained for 2 h in 50 μg ml^− 1^ PI, and subsequently analyzed on a BD Biosciences (BDB) Dual Laser FACSalibur (Becton Dickinson, Franklin Lakes, NJ, USA) flow cytometer, using the forward (FS) and side (SS) scattering, together with the red PI fluorescence (> 670 nm) detected by the FL3 detector. Data were generated at low flow rate (~ 1000 nuclei/min). Data analysis was performed using the BD FACSDiva 4.0 software (BD Biosciences, San José, CA, USA). Individual nuclei were gated from aggregates and debris by their area (FL3-A) vs. width (FL3-W) fluorescence signal. Measures were obtained from a minimum of 10,000 nuclei per sample. Genome sizes were estimated using the formula: GS_*D. subobscura* (*ch-cu*)_ = GS_*D. virilis*_ × (PI–fluor _*D. subobscura* (*ch-cu*)_) / (PI–Fluor_*d. virilis*_).

### High molecular weight genomic DNA isolation and PacBio whole-genome sequencing

High-quality high molecular weight gDNA was obtained from 60 mg mixes of − 80 °C frozen adult males and females, using a modified version of the phenol/chloroform method of Chen et al., [103] that yields ~ 25 μg of high quality DNA per assay, as assessed by NanoDrop ND1000 (NanoDrop Technologies Inc., Wilmington, DE, USA) spectrophotometer and standard agarose gel electrophoresis. The genome of the inbred *ch-cu* line was sequenced to nominal 40-fold genome coverage using PacBio (Pacific Biosciences, Menlo Park, CA, USA) RSII single-molecule real-time (SMRT) technology from a 20-kb SMRTbell template library, using P6-C4 chemistry and seven SMRT cells. Libraries construction and PacBio sequencing were outsourced to Macrogen (Macrogen Inc., Seoul, South Korea).

### De novo genome assembly

Raw PacBio reads were assembled using the Canu assembler (Ver. 1.5 [104]) on recommended settings for read error correction, trimming and assembly, and genome size set at 150 Mb (see below). In addition, we also tried HINGE [105], FALCON [106] and MECAT [107]. Compared to Canu, these alternative bioinformatics pipelines produced smaller and less contiguous assemblies on our data. These analyses were performed on a 2.80-GHz 8-CPU Intel Xeon 64-bit 32 GB-RAM computer running Ubuntu 16.04 LTS.

### Genome scaffolding

Chromosomal assignment, ordering and orientation of Canu contigs was accomplished in four steps. In step I, the contigs were checked for the presence of inter- and intra-chromosomal chimeras using a semi-automatic recursive approach combining: i) cross-species synteny information inferred using BLAT [108] and BLASTN [109], setting the genome of *D. melanogaster* (release r6.22) and the more closely related, yet not so-well characterized genome of *D. pseudoobscura* (r3.04) as the reference. Here, BLASTN was used in relatively few cases where BLAT either did not return a hit, returned multiple equal score hits, or returned a hit to scaffold unknown from *D. pseudoobscura*. The first of these three cases involved short and fast-evolving contigs and bacterial contigs; the second one involved contigs containing Repbase (Ver. 20,150,897 [110]) identified repetitive sequences, which were re-examined after masking of the repeats; in the third case, BLASTN was used to confirm that the target contig mapped exclusively to one scaffold. Cross-species synteny information obtained in this way was combined with ii) the wealth of available *D. subobscura*’s physical mapping [18, 84, 111] and genetic linkage [13, 112, 113] data. Markers’ sequences were retrieved from FlyBase 2.0 (release FB2017_02) using gene names and/or annotation symbols provided by the authors. In step II, Canu contigs that passed step I were scaffolded using SSPACE-LongRead (Ver. 1–1 [73]). In step III, the resulting SSPACE scaffolds were submitted to a second round of quality check as in step I. In step IV, the assembled sequence that passed step III was assigned genomic coordinates based on the physical location of the markers.

### Genome annotation

Prediction and annotation of the genome assembly was conducted using MAKER (Ver. 3.01.02. -beta [114, 115]) annotation pipeline with default parameters. Repetitive elements were identified using RepeatMasker (Ver. 4.0.6 [116]) combined with the *Drosophila* genus specific repeat library included in Repbase database. Two previously described satellites, namely *sat290* [90] and *SGM-sat* [91] were absent from the Repbase database, thereby they were ascertained separately by BLAST search using already available sequences from the *D. guanche sat290* [90] and the *D. subobscura SGM*-*sat* (GenBank accession AF043638.1 [91]) as queries. SNAP [117], AUGUSTUS [118], GeneMark-ES [119], and geneid [120] were selected for ab initio gene model prediction on the repeat masked genome sequence. Proteomes from 12 *Drosophila* species from Flybase database (FB2017_05, released October 25, 2017 [121]), and additional 491 *D. subobscura* protein sequences from UniProt database (release 2017_12 [122]) were used in the analysis.

The quality of the annotation was controlled using the Annotation Edit Distance (AED) metric [123]. AED values are bounded between 0 and 1; an AED value of 0 indicates perfect agreement of the annotation to aligned evidence. Conversely, a value of 1 indicated no evidence support.

Functional annotation of MAKER-predicted proteins was made by BLASTP (Ver. 2.6.0+) searches against the *Drosophila* UniProt-SwissProt manually curated datasets [124]. Prediction of protein functional domains was accomplished using InterProScan (Ver. 5.29–68.0 [125]) on the Pfam [126], InterPro [127], and Gene Ontology (GO) [128, 129] domain databases. UniProt-SwissProt BLAST and InterProScan functional assignments were extracted using the ANNotation Information Extractor (ANNIE [130]), which assigns gene names and products by database cross-referencing. InterProScan functional assignments were mapped to Gene Ontology (GO) terms using Blast2GO (Ver. 5.0.13 [131]). The combined graph function of Blast2GO was used to generate gene ontology graphs and pie charts from the GO terms.

Genome assembly and annotation completeness was gauged using the Benchmarking Universal Single-Copy Orthologs (BUSCO) tool (BUSCO, Ver. 3 [132]) analysis against the diptera_odb9 dataset, which contains 2799 highly-conserved, single-copy genes likely to be present in any dipteran genome. The dipteran set was selected, because being the most narrowly defined *Drosophila*-including set, it is also the largest, therefore the one expected to provide the best resolution.

### Mitogenome assembly and annotation

Annotation of the *D. subobscura* mitogenome was conducted using the MITOS online tool (http://mitos.bioinf.uni-lipzig.de/index.py [133]), with default settings, metazoan reference, and invertebrate genetic code, and was further adjusted manually according to its alignment with available mitogenomes from other *Drosophila* species.

### Orthologous group assignment and gene family expansion/contraction analyses

The complete set of *D. subobscura* annotated proteins were clustered into orthologous groups by comparison with the 12 *Drosophila* genomes (FlyBase releases *dana_R1.06*, *dere_R1.05*, *dgri_R1.05*, *dmel_R6.22*, *dmoj_R1.04*, *dper_R1.3*, *dpse_R3.04*, *dsec_R1.3*, *dsim_R2.02*, *dvir_R1.07*, *dwil_R1.05*, and *dyak_R1.05*), plus that of its close relative *D. guanche* (*dgua_R1.0* [59]). Orthologous group assignment was conducted using OrthoMCL (Ver. 5 [134]) on default settings. OrthoMCL generates orthologous groups via all-to-all BLASTP comparison followed by Markov clustering of the reciprocal best similarity pairs.

Analysis of gene family expansion and contraction was conducted using the Computational Analysis of gene Family Evolution (CAFE Ver. 3.1 [92]) tool. For a specified ultrametric phylogenetic tree, and given the gene family sizes in the extant species, CAFE uses a maximum likelihood stochastic birth-and-death process to model the rate and direction of change in gene family size (in number of gene births and deaths per gene per million years; symbolized λ) over the tree. CAFE was run on default parameters using a 14 species ultrametric tree built by grafting *D. subobscura* and *D. guanche* onto the 12 *Drosophila* tree used by Hahn et al. [135] at positions obtained from the *TimeTree* database (http://www.timetree.org/ [136]):

(((((((*Dsim*:2.1,*Dsec*:2.1):3.2,*Dmel*:5.3):5.9,(*Dere*:8.5,*Dyak*:8.5):2.7):42.1,*Dana*:53.3):2.3,((*Dpse*:1.4,*Dper*:1.4):13.1,(*Dsub*:3.1,*Dgua*:3.1):11.4):41.1):6.8,*Dwil*:62.4):0.8,((*Dvir*:32.7,*Dmoj*:32.7):4.3,*Dgri*:37):26.2); with branch lengths given in million years.

Model-fitting considered three nested likelihood models of gene family size evolution. The first model assumes a single global λ_G_ for all lineages. The second model allows for three λ to accommodate for fast- (λ_F_ ≥ 0.010), medium- (0.010 > λ_M_ > 0.002), and slow-evolving (λ_S_ ≤ 0.002) branches. Assignment of each branch to its corresponding λ category (i.e., λ_F_, λ_M_ or λ_S_) in this model was made a priori, based on the best results for a fully 26 λ-parameters model (i.e., one for each branch of the phylogeny), as in Hahn et al. [135]. The third model is a five λ generalization of the second model to allow for the terminal branches leading to *D. subobscura* and *D. guanche* having their own rates (i.e., λ_Ds_ and λ_Dg_, respectively). Estimates of λ obtained using this approach are sensitive to suboptimal genome assembly and/or annotation. Therefore, the obtained best-fitting model was refined by adding to it a term of error (ε) in genome quality. The effect of the error term on λ provides an indirect measure of genome assembly and/or annotation completeness [92]. For each model, at least five CAFE runs were performed and those runs with the highest likelihood score per model were included. To meet the CAFE assumption that gene families must have been present at the root of the tree, only families found in at least one species of both the *Sophophora* and *Drosophila* subgenera, were considered. Both OrthoMCL and CAFE analyses were conducted considering only the longest splice forms.

Functional enrichment analyses of gene families uncovered to have been rapidly evolving along the terminal branch of this species by CAFE were carried out using the Blast2GO [131] implementation of the one-sided Fisher’s exact test, with false discovery rate (FDR) < 0.001. Enriched GO terms were summarized and visualized using the online version of REVIGO (http://revigo.irb.hr/ [137]). This tool identifies representative GO terms by semantic similarity.

### Whole-genome synteny analysis

The genome of *D. subobscura* was analyzed for conservation of synteny against those of three increasingly distant species, namely *D. guanche* (*dgua_R1.01* [59]), *D. pseudoobscura* (*dpse_R3.04*), and *D. melanogaster* (*dmel_R6.22*), using the Synteny Mapping and Analysis Program (SyMAP, Ver. 4.2. [138, 139]) tool on default options. SyMAP is a long-range whole-genome synteny mapping tool devised to accommodate for intervening micro-rearrangements which could result from misassembling, but also from real structural changes. Therefore, SyMAP seemed especially suited for investigating large, cytologically visible recent chromosomal rearrangement events that are the focus of the present study.

## Additional files

Additional file 1: **Table S1**. Genome sequencing and assembly statistics (coverages based on a 150Mb genome size; lengths are in bp). (DOCX 43 kb)

Additional file 2: **Table S2**. Genetic markers used for validation, and physical anchoring, ordering and orientation of scaffolds. In total, 683 markers were considered, of which 621 were used. 62 markers were not used because they showed inconsistencies as to their localization with respect to markers from other studies and our own data. The MS Excel file contains eight spreadsheets, including one for this title, one for each of the five major pseudochromosomes (i.e., A, J, U, E and O), one with a summary, and one with the references for the marker data. For each pseudochromosome, markers are listed in column “A”, including used cytological (numbered, black), used linkage (numbered, blue), and nonused (nonnumbered, red) markers. For each marker, information relative to its name, cytological localization, authors, corresponding *D. pseudoobscura* “GA” gene model name, inconsistency where it applies, coordinates in the pseudochromosome, scaffold name, scaffold orientation and cytological span, and BLASTn statistics is provided in subsequent columns, from “B” to “Y”. Column “X” provides the number of used marker per scaffold. Alternating color in the background denotes different scaffolds. Cytological coordinates are always relative to the Kunze-Mühl and Müller [12] standard reference map. From the summary spreadsheet, most of the inconsistencies (72%) come from one (Laayouni et al. 2007) out of the total 26 cited works. Excluding that study, the total percent of inconsistencies is only 2.85% (i.e, 17 out of 638 markers). (XLSX 168 kb)

Additional file 3: **Figure S1**. RelTime timetree of 14 *Drosophila* species obtained using the maximum-likelihood tree-topology that results after GTR + G + I best-fit modeling of a 50 concatenated nuclear low-codon bias orthologous gene alignment dataset. Blue diamonds indicate Obbard et al. [78] mutation-based calibrated nodes, and orange boxes 95% confidence intervals for target divergences. (PDF 10 kb)

Additional file 4: **Table S3**. *D. subobscura* mitogenome gene content and order (lengths in bp)*.* (DOCX 45 kb)

Additional file 5: **Table S4**. Repetitive content of the *D. subobscura* genome. (DOCX 43 kb)

Additional file 6: **Figure S2**. OrthoMCL analysis of gene families in *D. subobscura*. Numbers of orthoMCL clusters and of genes within those clusters on each node are given in black and white rectangles, respectively. (PDF 15 kb)

Additional file 7: **Table S5**. Optimal CAFE model selection for the evolution of gene family size along the 14 *Drosophila* ultrametric tree in Figures S3-S4. Shown are the four assayed increasingly complex models, including the 1-λ and 3-λ models, and the 5-λ model without and with global assembling error term (ε); and their corresponding parameter estimates, including global (λ_G_), slow (λ_S_), medium (λ_M_), fast (λ_F_), *D. subobscura* (λ_Ds_) and *D. guanche* (λ_Dg_) lambdas, and global error, and maximum-likelihood scores (−lnL). (DOCX 42 kb)

Additional file 8: **Figure S3**. CAFE analysis of the evolution of gene family size in *D. subobscura*. Shown on each branch are its corresponding numbers of expanded (left) and contracted (right) gene families. Circled numbers on nodes are identifiers for internal branches of the phylogeny leading to those nodes. The colors of the circles indicate estimated rates of gene gain and loss according to the legend on the upper left (blue: slow, grey: medium, red: fast). (PDF 33 kb)

Additional file 9: **Figure S4**. CAFE analysis of the evolution of gene family size in *D. subobscura*. Shown on each branch are its corresponding numbers of significantly expanded (green) and contracted (orange) gene families. Circled numbers on nodes are identifiers for internal branches of the phylogeny leading to those nodes. The colors of the circles indicate estimated rates of gene gain and loss according to the legend on the upper left (blue: slow, grey: medium, red: fast). (PDF 33 kb)

Additional file 10: **Table S6**. Over represented GO Terms among CAFE significantly expanded gene families in *D. subobscura* inferred using one-sided Fisher exact test (FDR < 0.001) implemented in Blast2Go (BP: Biological Process; MF: Molecular Function; CC: Cellular Component). (DOCX 46 kb)

Additional file 11: **Table S7**. Over represented GO Terms among CAFE significantly contracted gene families in *D. subobscura* inferred using one-sided Fisher exact test (FDR < 0.001) implemented in Blast2Go (BP: Biological Process; MF: Molecular Function; CC: Cellular Component). (DOCX 47 kb)

Additional file 12: **Figure S5**. REVIGO summary scatterplot for 27 over-represented Biological Process GO terms in CAFE-expanded gene families. Shown GO term names denote cluster representatives centered on their corresponding GO term. Distances between GO terms are in units of semantic similarity. Circle color indicates FDR values, and circle size generality of the GO term (the lower, the greater the uniqueness of the term). (PDF 88 kb)

Additional file 13: **Figure S6**. REVIGO summary scatterplot for 17 over-represented Molecular Function GO terms in CAFE-expanded gene families. Shown GO term names denote cluster representatives centered on their corresponding GO term. Distances between GO terms are in units of semantic similarity. Circle color indicates FDR values, and circle size generality of the GO term (the lower, the greater the uniqueness of the term). (PDF 90 kb)

Additional file 14: **Figure S7**. REVIGO summary scatterplot for 9 over-represented Cellular Component GO terms in CAFE-expanded gene families. Shown GO term names denote cluster representatives centered on their corresponding GO term. Distances between GO terms are in units of semantic similarity. Circle color indicates FDR values, and circle size generality of the GO term (the lower, the greater the uniqueness of the term). (PDF 49 kb)

Additional file 15: **Figure S8**. REVIGO summary scatterplot for 51 over-represented Biological Process GO terms in CAFE-contracted gene families. Shown GO term names denote cluster representatives centered on their corresponding GO term. Distances between GO terms are in units of semantic similarity. Circle color indicates FDR values, and circle size generality of the GO term (the lower, the greater the uniqueness of the term). (PDF 126 kb)

Additional file 16: **Figure S9**. REVIGO summary scatterplot for 12 over-represented Molecular Function GO terms in CAFE-contracted gene families. Shown GO term names denote cluster representatives centered on their corresponding GO term. Distances between GO terms are in units of semantic similarity. Circle color indicates FDR values, and circle size generality of the GO term (the lower, the greater the uniqueness of the term). (PDF 90 kb)

Additional file 17: **Figure S10**. REVIGO summary scatterplot for 8 over-represented Cellular Component GO terms in CAFE-contracted gene families. Shown GO term names denote cluster representatives centered on their corresponding GO term. Distances between GO terms are in units of semantic similarity. Circle color indicates FDR values, and circle size generality of the GO term (the lower, the greater the uniqueness of the term). (PDF 63 kb)

Additional file 18: **Table S8**. Number of syntenic blocks between *D. subobscura* and increasingly distant relatives. (DOCX 41 kb)

Additional file 19: **Table S9**. Average size of the syntenic block (in Mb) between *D. subobscura* and increasingly distant relatives. (DOCX 41 kb)

Additional file 20: **Figure S11**. Schematic of the strategy used for inversion breakpoint detection. From top to bottom: shown are (a) two noninverted (SB1 and SB3; pink) and one inverted (SB2; green) hypothetical SyMAP synteny blocks between two taxa (1 and 2). The regions flanking the points of broken synteny (vertical dotted lines) are labelled A-D correspondingly; (b) BLASTing regions AB and CD from taxon 1 against the genome of taxon 2 each produces two hits (c) at opposite ends of the inverted synteny block with associated overhangs; (d) steps b-c are repeated using taxon 2 for the BLAST queries to test for reciprocal consistency (see main text for more detail). (PDF 97 kb)

Additional file 21: **Table S10**. Synteny analysis of inversion breakpoints. Provided is breakpoint information for 12 inversions, including six from pseudochromosome A (h1, h2, h3, h4, 5 and 6), one from J (ST), two from U (1 and 2), two from E (g1 and ST), and one from O (ms). The MS Excel file contains six spreadsheets, including one for this title, and one for each of the five major pseudochromosomes (i.e., A, J, U, E and O). For each pseudochromosome, inversions are listed in column “A”. For each inversion, information about the three protein coding genes flanking each side of each breakpoint in three species, including *D. melanogaster*, *D. guanche* and *D. subobscura* is provided in subsequent columns, from “B” to “Q”. This information includes species names, names and pseudochromosome coordinates of the three coding gene markers on both sides of each distal and proximal breakpoint, and the size of the pseudochromosome segment spanned by the breakpoints in Mb. Also provided is, for each breakpoint, its cytological and estimated pseudochromosome coordinates, and its hypothetical originating mechanism with the length of the associated duplication where it applies. Cells color background indicate contiguity (brown) or altered (yellow) order of the markers relative to the outgroup (*D. melanogaster*/*D. pseudoobscura*). For example, in the case of hypothetical inversion 1 of the A chromosome (i.e., h1) in *D. subobscura*, the three markers downstream the proximal breakpoint and upstream the distal breakpoint are in reverse order relative to *D. guanche*, which shows the markers ordered as in *D. melanogaster*. Reciprocal BLASTn searches with each breakpoint did not detect evidence of duplication, suggesting that the most likely originating mechanism of inversion A_h1_ (depicted in yellow) is simple, or nearly straight breaks. (XLSX 31 kb)
