# Supplementary material for: Long-read based assembly and synteny analysis of a reference Drosophila subobscura genome reveals signatures of structural evolution driven by inversions recombination-suppression effects
Source: BMC Genomics. 2019 Mar 18;20:223. doi: 10.1186/s12864-019-5590-8 (PMC6423853; doi:10.1186/s12864-019-5590-8)
Supplement: Supplementary file 1 — Table S1. Genome sequencing and assembly statistics (coverages based on a 150Mb genome size; lengths are in bp). (DOCX 43 kb) [file 12864_2019_5590_MOESM1_ESM.docx]

**Table S1.** Genome sequencing and assembly statistics (coverages based on a 150Mb genome size; lengths are in bp).

| **PacBio sequencing statistics** |  |
| --- | --- |
| Reads | 1,252,701 |
| Avg. read length | 8,003 |
| Longest read | 52,567 |
| Bases | 10,025,366,103 |
| Bases per SMRT cell | 1,432,195,158 |
| Genome coverage | 66.8× |
| **Canu assembly statistics** |  |
| Reads > 1kb | 1,183,935 |
| Bases in reads > 1kb | 9,990,943,511 |
| Corrected reads | 939,323 |
| Bases in corrected reads | 7,881,319,324 |
| Trimmed reads | 1,060,943 |
| Bases in trimmed reads | 6,474,536,519 |
| Avg. trimmed read length | 6,103 |
| Longest trimmed read | 32,866 |
| Genome coverage | 43.2× |
| Contigs | 327 |
| Bases in contigs | 135,636,516 |
| **Final assembly statistics** |  |
| Contigs | 212 |
| Bases in contigs | 129,182,992 |
| Scaffolds | 186 |
| Bases in scaffolds | 129,236,726 |
| L50 | 7 |
| N50 | 5,954,457 |
| Unknown scaffolds | 33 |
| Bases in unknown scaffolds | 823,561 |
| Chromosome-assigned scaffolds | 153 |
| Bases in chromosome-assigned scaffolds | 128,413,165 |
| Assigned-only scaffolds | 90 |
| Bases in assigned-only scaffolds | 3,551,346 |
| rDNA scaffolds | 19 |
| Bases in rDNA scaffolds | 862,921 |
| Non rDNA scaffolds | 71 |
| Bases in non rDNA scaffolds | 2,688,425 |
| Pseudochromosome-scaffolds | 63 |
| Bases in pseudochromosome-scaffolds | 124,861,819 |
|  |  |
