# Supplementary figures and images for: Long-read based assembly and synteny analysis of a reference Drosophila subobscura genome reveals signatures of structural evolution driven by inversions recombination-suppression effects
Source: BMC Genomics. 2019 Mar 18;20:223. doi: 10.1186/s12864-019-5590-8 (PMC6423853; doi:10.1186/s12864-019-5590-8)

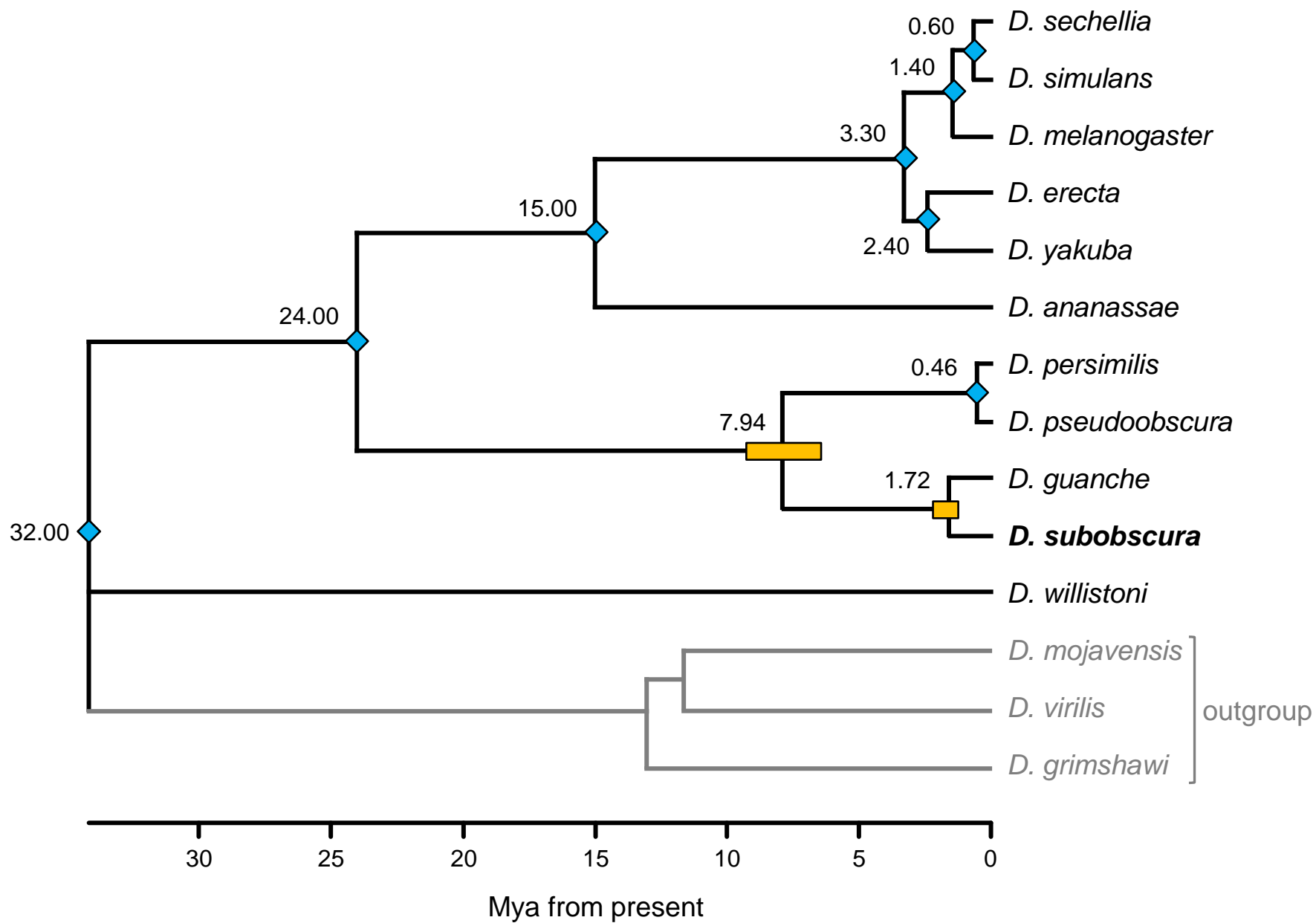

Supplement: Supplementary file 3 — Figure S1. RelTime timetree of 14 Drosophila species obtained using the maximum-likelihood tree-topology that results after GTR + G + I best-fit modeling of a 50 concatenated nuclear low-codon bias orthologous gene alignment dataset. Blue diamonds indicate Obbard et al. [78] mutation-based calibrated nodes, and orange boxes 95% confidence intervals for target divergences. (PDF 10 kb) [file 12864_2019_5590_MOESM3_ESM.pdf]

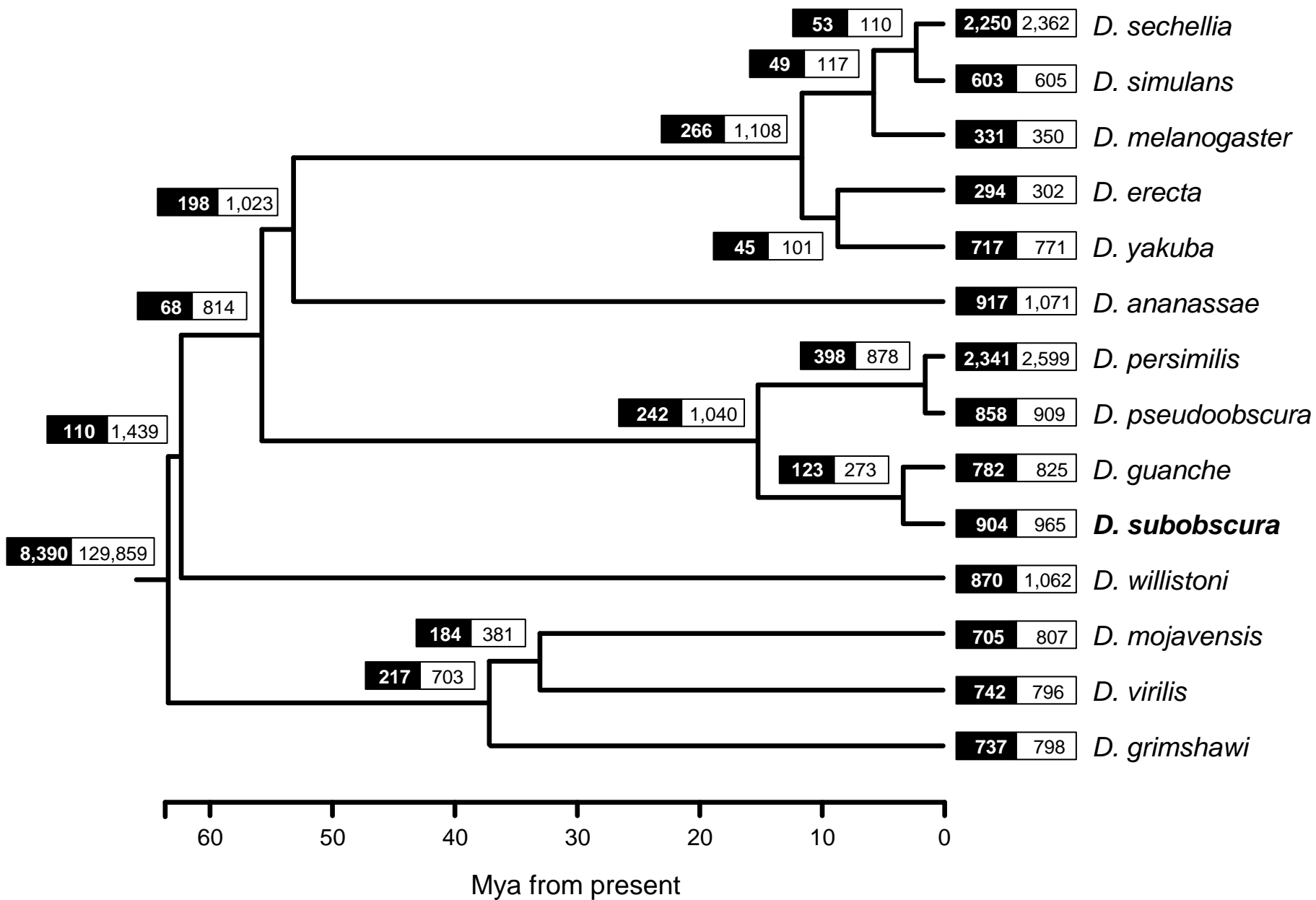

Supplement: Supplementary file 6 — Figure S2. OrthoMCL analysis of gene families in D. subobscura. Numbers of orthoMCL clusters and of genes within those clusters on each node are given in black and white rectangles, respectively. (PDF 15 kb) [file 12864_2019_5590_MOESM6_ESM.pdf]

$\lambda = 0.00085$   
 $\lambda = 0.00204$   
 $\lambda = 0.01000$

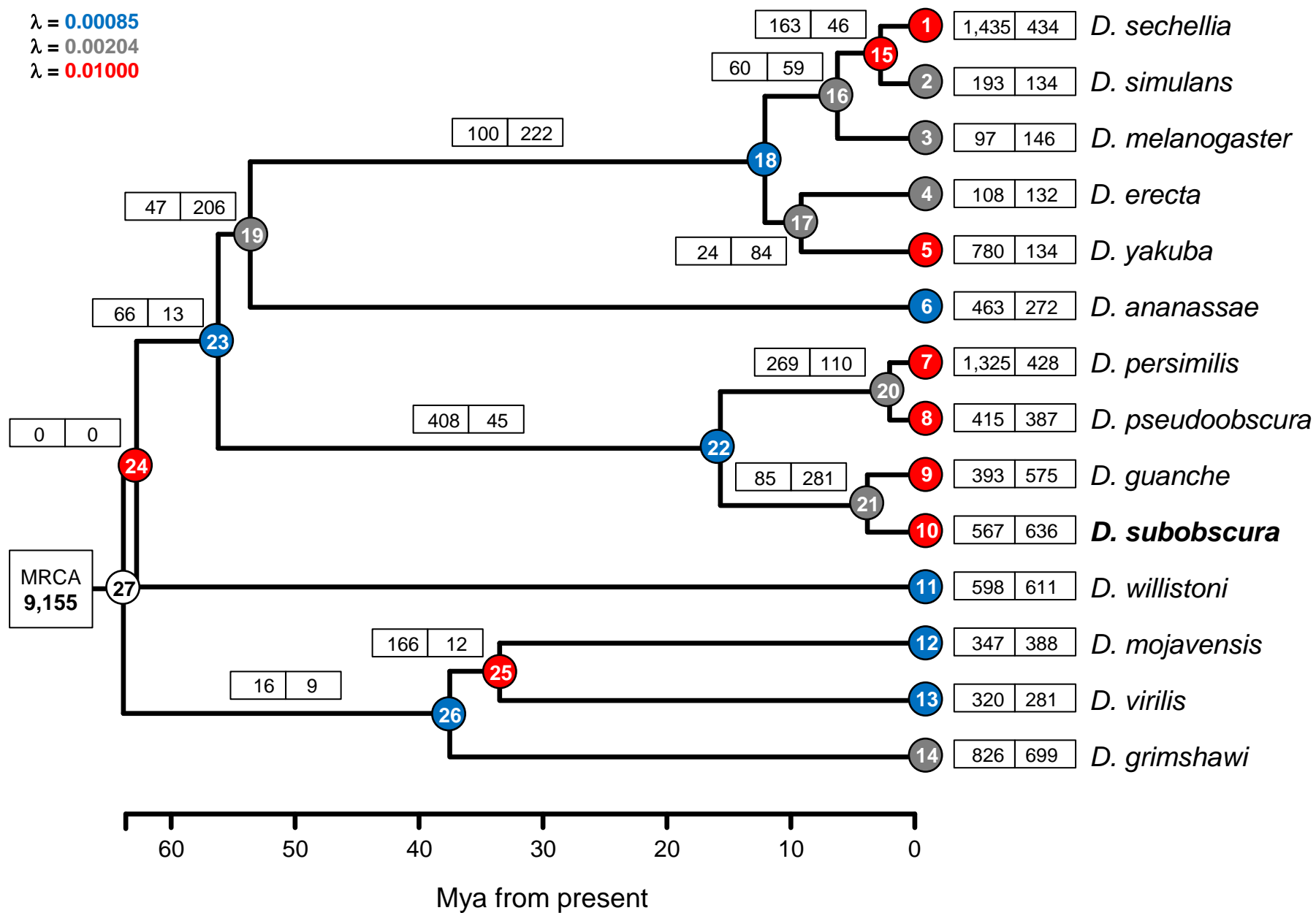

Supplement: Supplementary file 8 — Figure S3. CAFE analysis of the evolution of gene family size in D. subobscura. Shown on each branch are its corresponding numbers of expanded (left) and contracted (right) gene families. Circled numbers on nodes are identifiers for internal branches of the phylogeny leading to those nodes. The colors of the circles indicate estimated rates of gene gain and loss according to the legend on the upper left (blue: slow, grey: medium, red: fast). (PDF 33 kb) [file 12864_2019_5590_MOESM8_ESM.pdf]

$\lambda = 0.00085$   
 $\lambda = 0.00204$   
 $\lambda = 0.01000$

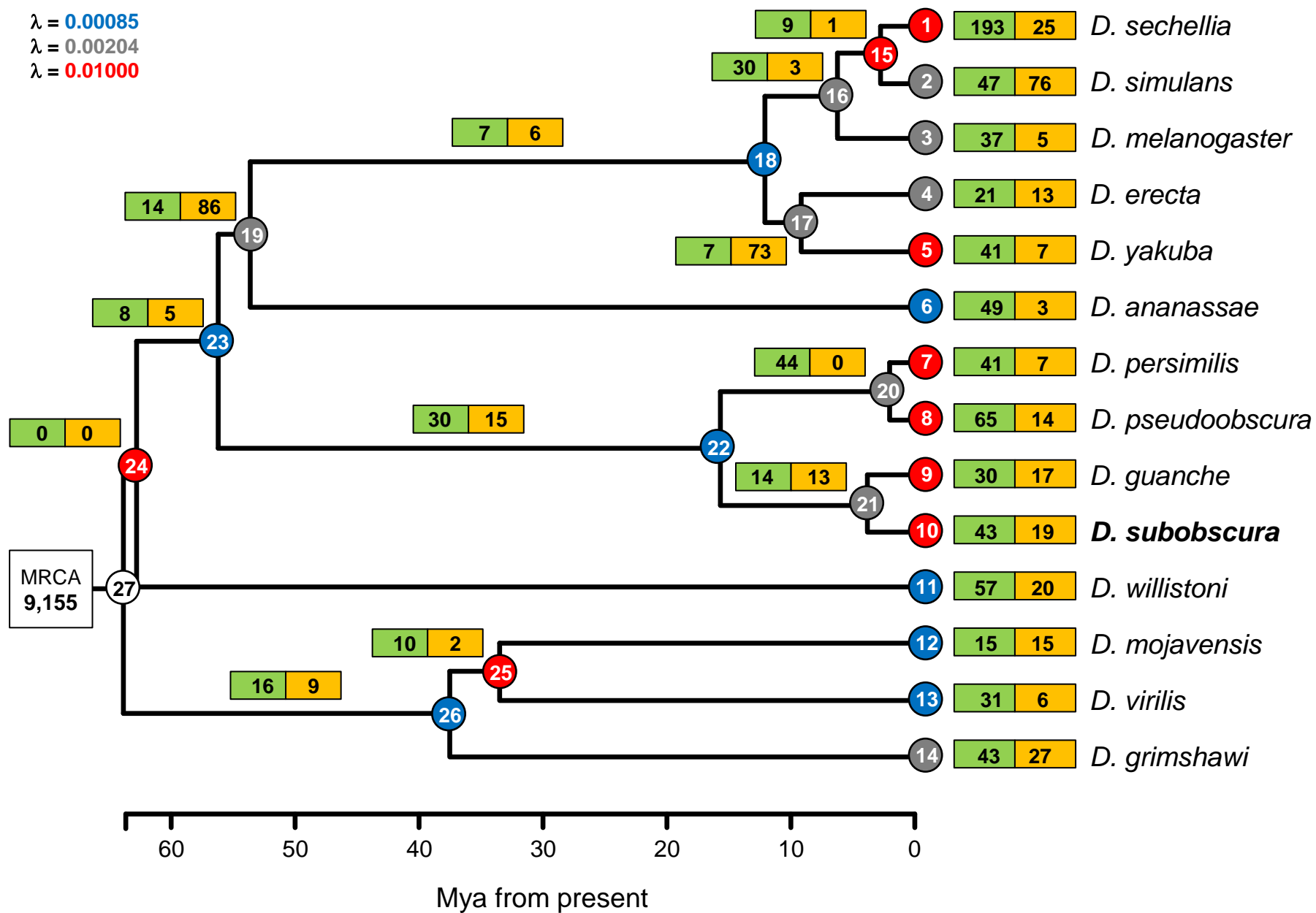

Supplement: Supplementary file 9 — Figure S4. CAFE analysis of the evolution of gene family size in D. subobscura. Shown on each branch are its corresponding numbers of significantly expanded (green) and contracted (orange) gene families. Circled numbers on nodes are identifiers for internal branches of the phylogeny leading to those nodes. The colors of the circles indicate estimated rates of gene gain and loss according to the legend on the upper left (blue: slow, grey: medium, red: fast). (PDF 33 kb) [file 12864_2019_5590_MOESM9_ESM.pdf]

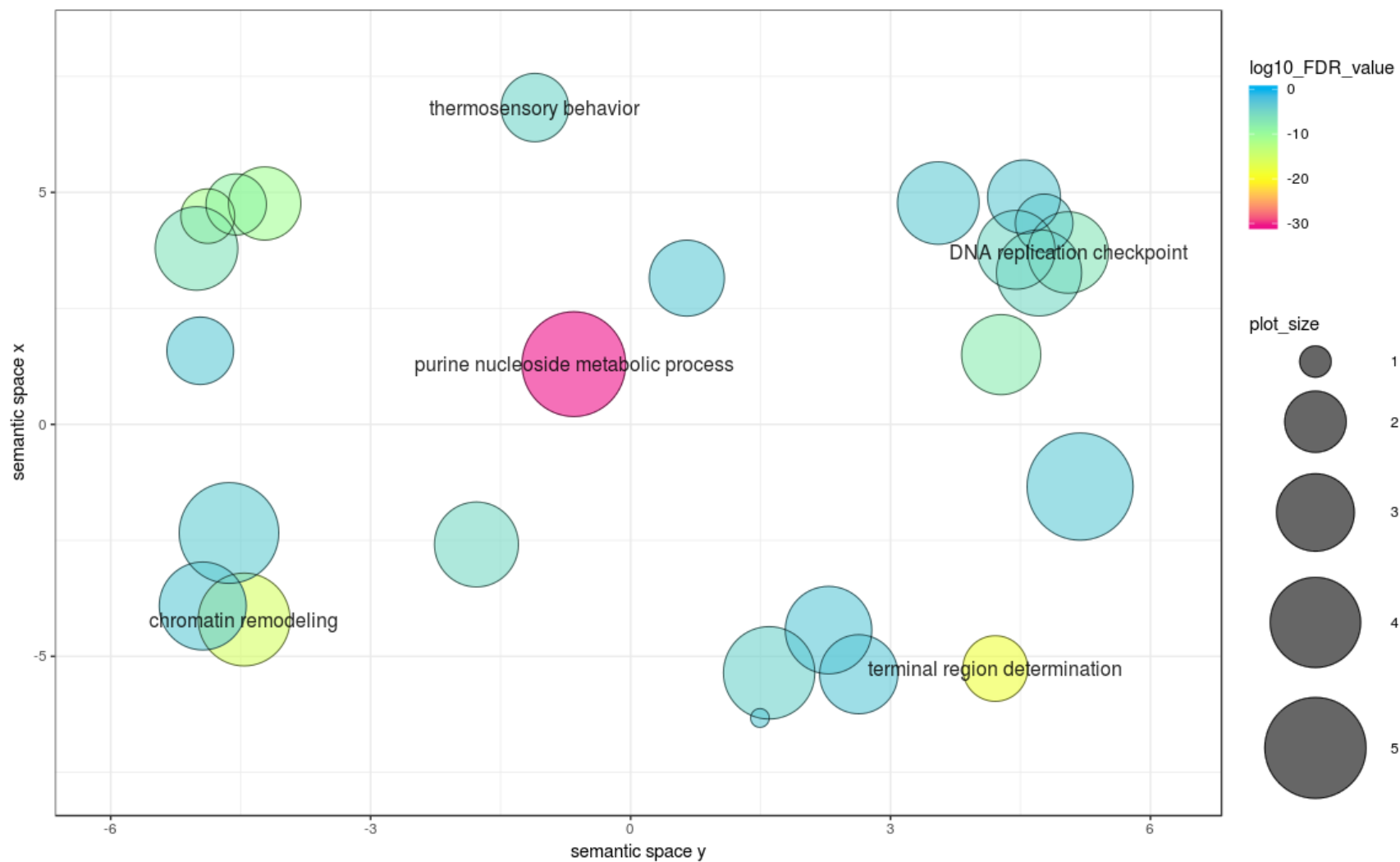

Supplement: Supplementary file 12 — Figure S5. REVIGO summary scatterplot for 27 over-represented Biological Process GO terms in CAFE-expanded gene families. Shown GO term names denote cluster representatives centered on their corresponding GO term. Distances between GO terms are in units of semantic similarity. Circle color indicates FDR values, and circle size generality of the GO term (the lower, the greater the uniqueness of the term). (PDF 88 kb) [file 12864_2019_5590_MOESM12_ESM.pdf]

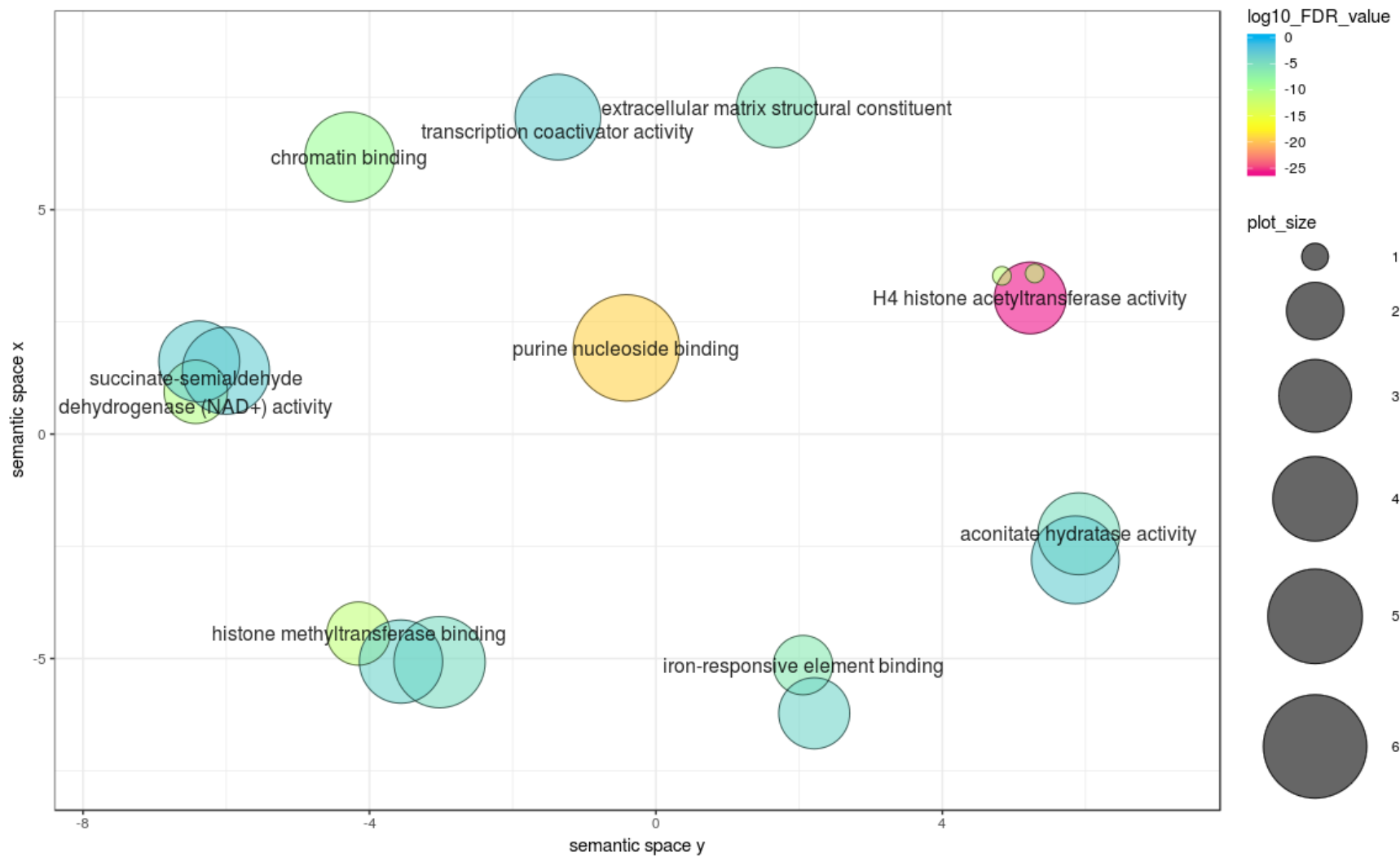

Supplement: Supplementary file 13 — Figure S6. REVIGO summary scatterplot for 17 over-represented Molecular Function GO terms in CAFE-expanded gene families. Shown GO term names denote cluster representatives centered on their corresponding GO term. Distances between GO terms are in units of semantic similarity. Circle color indicates FDR values, and circle size generality of the GO term (the lower, the greater the uniqueness of the term). (PDF 90 kb) [file 12864_2019_5590_MOESM13_ESM.pdf]

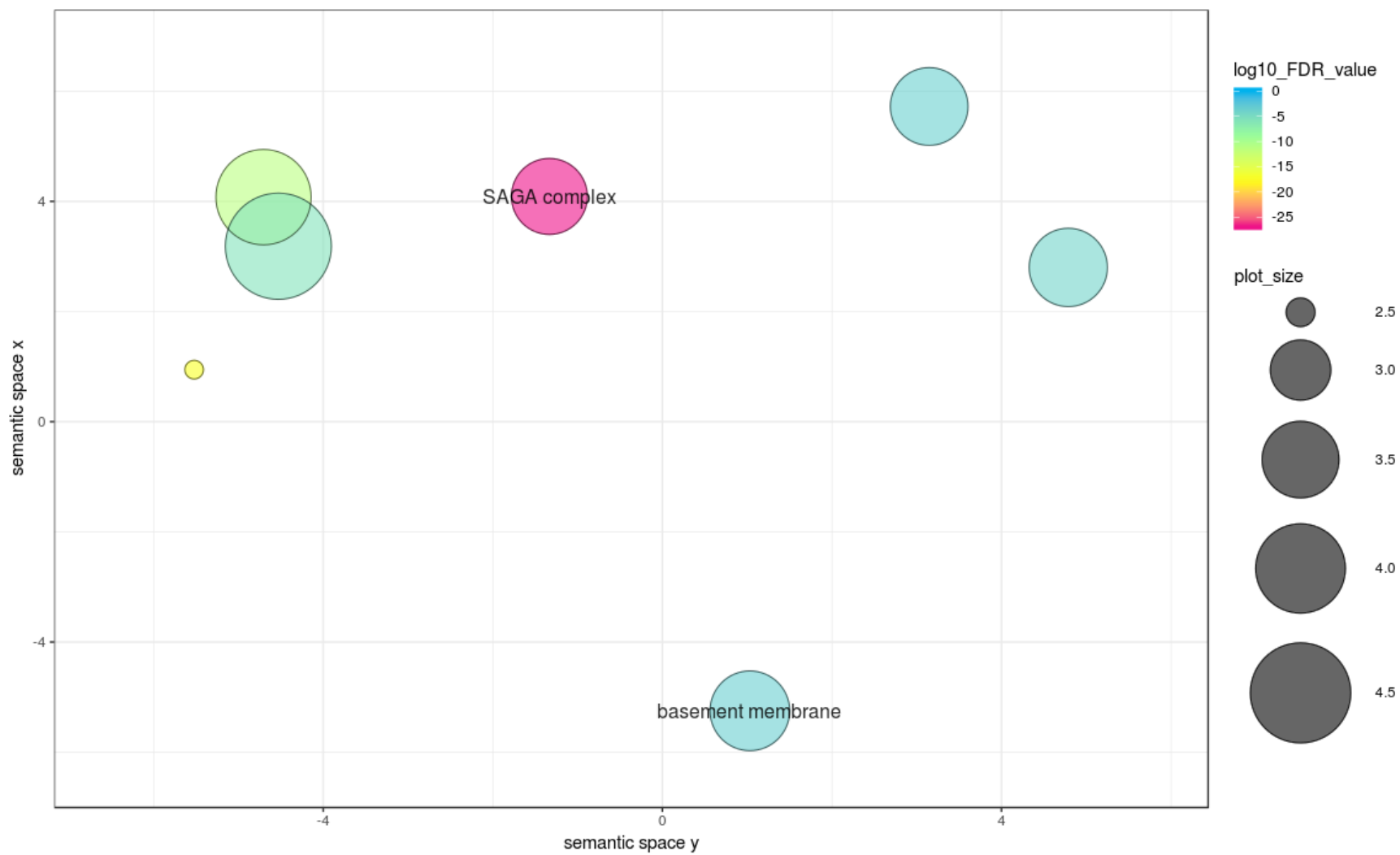

Supplement: Supplementary file 14 — Figure S7. REVIGO summary scatterplot for 9 over-represented Cellular Component GO terms in CAFE-expanded gene families. Shown GO term names denote cluster representatives centered on their corresponding GO term. Distances between GO terms are in units of semantic similarity. Circle color indicates FDR values, and circle size generality of the GO term (the lower, the greater the uniqueness of the term). (PDF 49 kb) [file 12864_2019_5590_MOESM14_ESM.pdf]

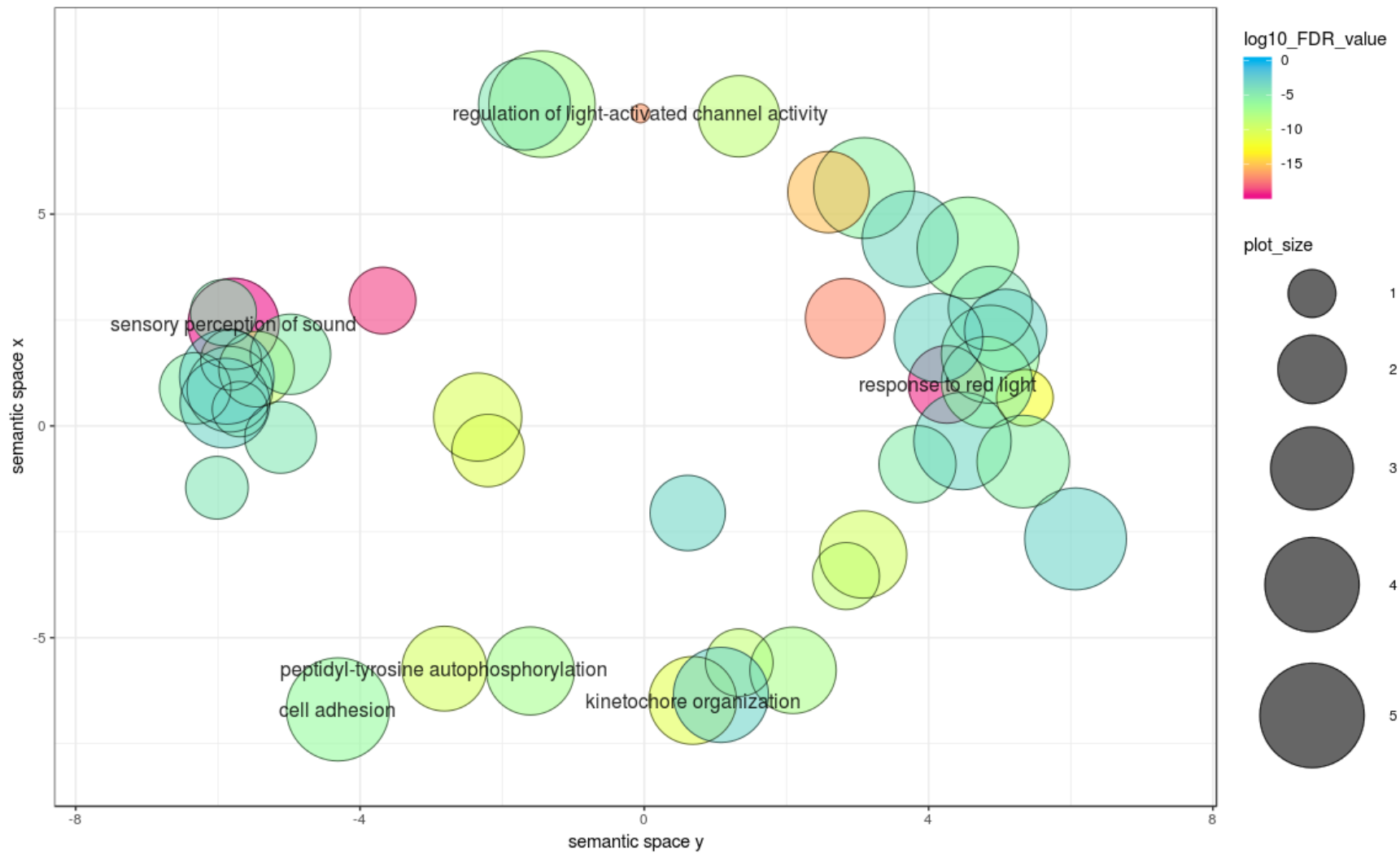

Supplement: Supplementary file 15 — Figure S8. REVIGO summary scatterplot for 51 over-represented Biological Process GO terms in CAFE-contracted gene families. Shown GO term names denote cluster representatives centered on their corresponding GO term. Distances between GO terms are in units of semantic similarity. Circle color indicates FDR values, and circle size generality of the GO term (the lower, the greater the uniqueness of the term). (PDF 126 kb) [file 12864_2019_5590_MOESM15_ESM.pdf]

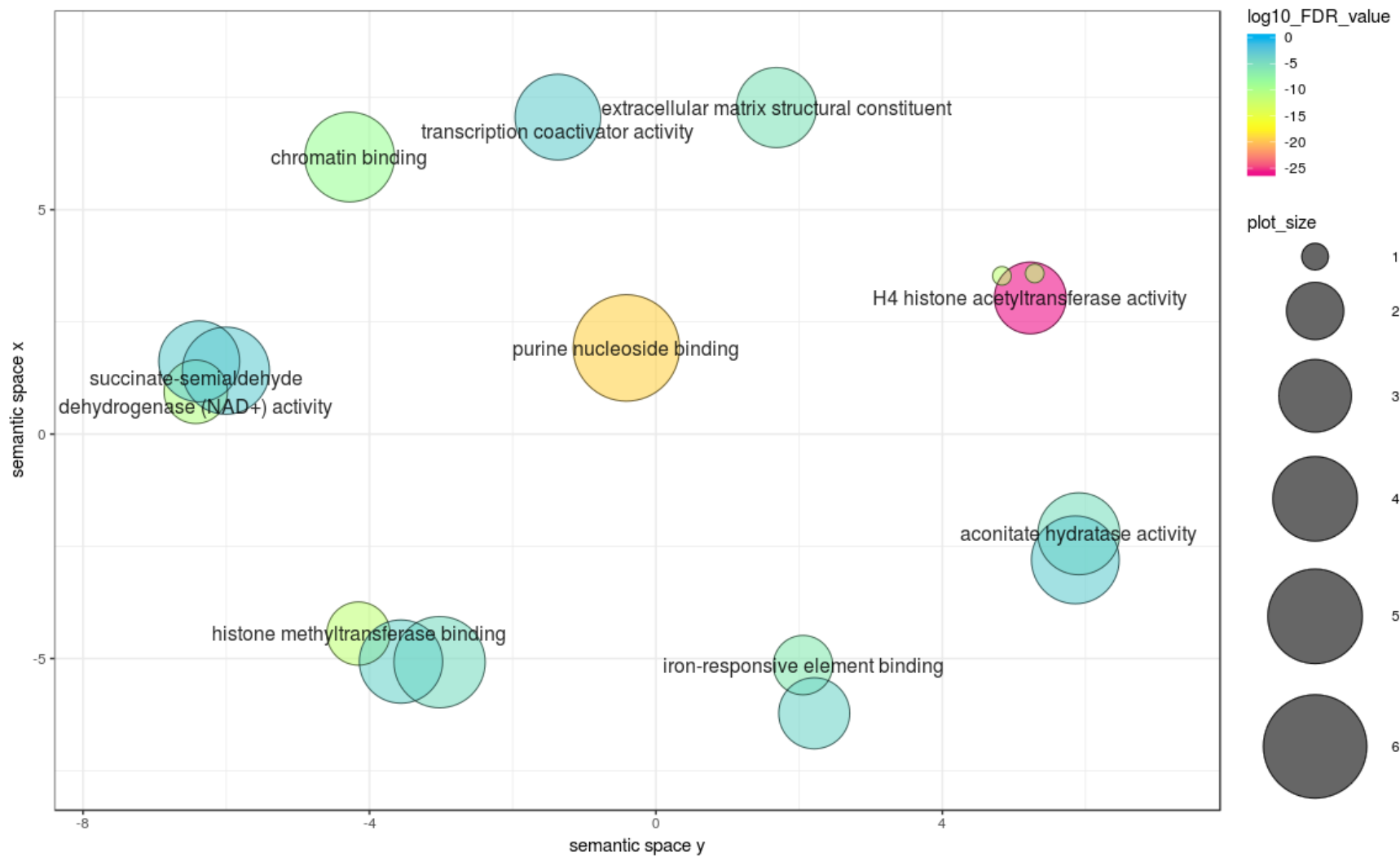

Supplement: Supplementary file 16 — Figure S9. REVIGO summary scatterplot for 12 over-represented Molecular Function GO terms in CAFE-contracted gene families. Shown GO term names denote cluster representatives centered on their corresponding GO term. Distances between GO terms are in units of semantic similarity. Circle color indicates FDR values, and circle size generality of the GO term (the lower, the greater the uniqueness of the term). (PDF 90 kb) [file 12864_2019_5590_MOESM16_ESM.pdf]

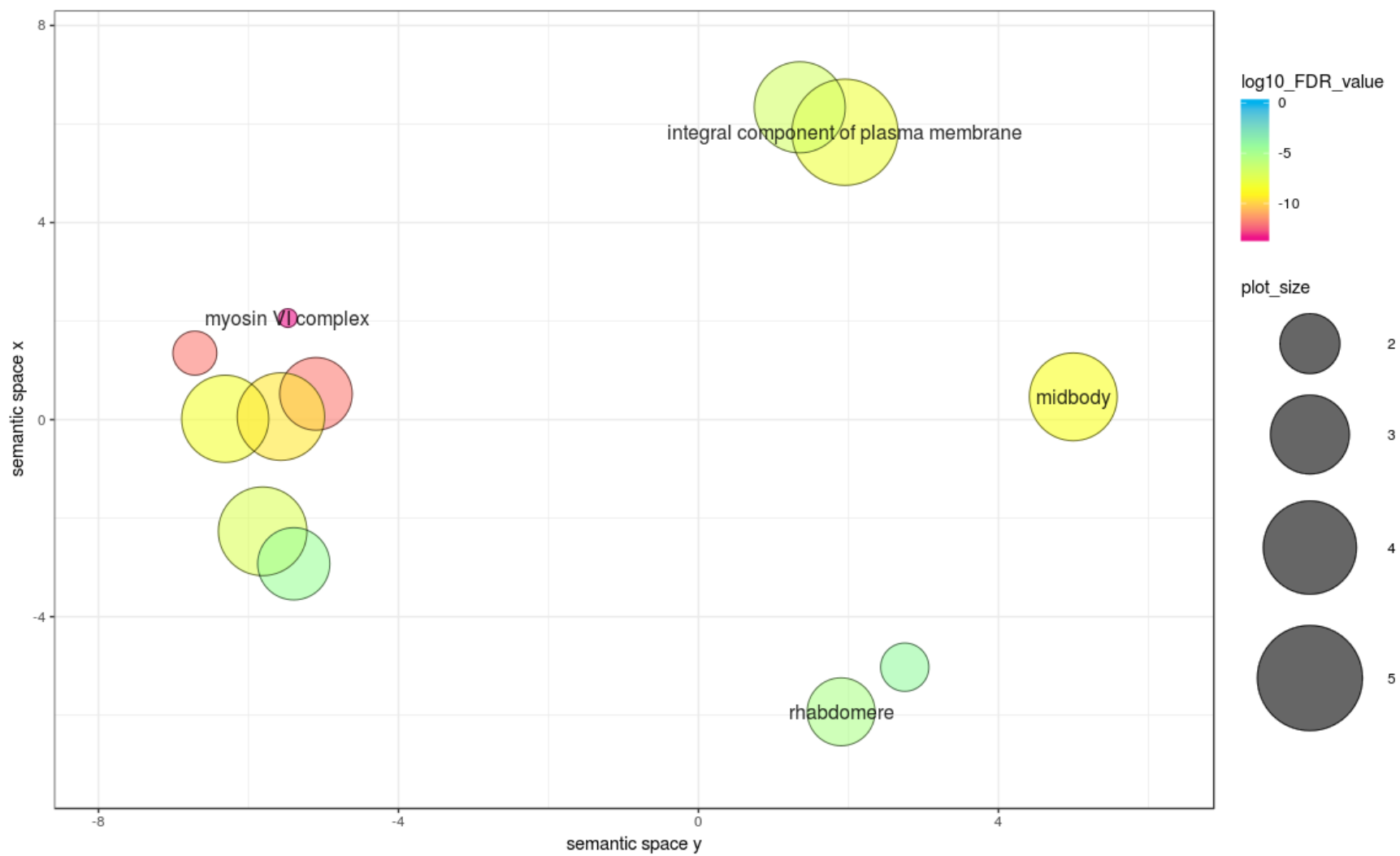

Supplement: Supplementary file 17 — Figure S10. REVIGO summary scatterplot for 8 over-represented Cellular Component GO terms in CAFE-contracted gene families. Shown GO term names denote cluster representatives centered on their corresponding GO term. Distances between GO terms are in units of semantic similarity. Circle color indicates FDR values, and circle size generality of the GO term (the lower, the greater the uniqueness of the term). (PDF 63 kb) [file 12864_2019_5590_MOESM17_ESM.pdf]
