# Supplementary material for: Long-read based assembly and synteny analysis of a reference Drosophila subobscura genome reveals signatures of structural evolution driven by inversions recombination-suppression effects
Source: BMC Genomics. 2019 Mar 18;20:223. doi: 10.1186/s12864-019-5590-8 (PMC6423853; doi:10.1186/s12864-019-5590-8)
Supplement: Supplementary file 4 — Table S3. D. subobscura mitogenome gene content and order (lengths in bp). (DOCX 45 kb) [file 12864_2019_5590_MOESM4_ESM.docx]

**Table S3.-** *D. subobscura* mitogenome gene content and order (lengths in bp)*.*

| **Annotation** | **Start** | **Stop** | **Length** | **Strand** |
| --- | --- | --- | --- | --- |
| tRNAI | 0 | 46 | 47 | + |
| tRNAQ | 84 | 152 | 69 | − |
| tRNAM | 152 | 220 | 69 | + |
| ND2 | 242 | 1236 | 995 | + |
| tRNAW | 1243 | 1308 | 66 | + |
| tRNAC | 1301 | 1363 | 63 | − |
| tRNAY | 1364 | 1429 | 66 | − |
| CoI | 1434 | 2942 | 1509 | + |
| tRNAL2 | 2966 | 3031 | 66 | + |
| CoII | 3037 | 3706 | 672 | + |
| tRNAK | 3723 | 3792 | 70 | + |
| tRNAD | 3793 | 3859 | 67 | + |
| ATP8 | 3886 | 4017 | 132 | + |
| ATP6 | 4012 | 4676 | 665 | + |
| CoIII | 4693 | 5470 | 777 | + |
| tRNAG | 5490 | 5553 | 64 | + |
| ND3 | 5554 | 5880 | 327 | + |
| tRNAA | 5906 | 5969 | 64 | + |
| tRNAR | 5971 | 6033 | 63 | + |
| tRNAN | 6034 | 6098 | 65 | + |
| tRNAS1 | 6099 | 6166 | 68 | + |
| tRNAE | 6167 | 6232 | 66 | + |
| tRNAF | 6251 | 6316 | 66 | − |
| ND5 | 6336 | 7955 | 1620 | − |
| tRNAH | 8052 | 8117 | 66 | − |
| ND4 | 8138 | 9457 | 1320 | − |
| ND4L | 9454 | 9714 | 261 | − |
| tRNAT | 9750 | 9814 | 65 | + |
| tRNAP | 9815 | 9880 | 66 | − |
| ND6 | 9895 | 10398 | 504 | + |
| COB | 10411 | 11517 | 1107 | + |
| tRNAS2 | 11550 | 11615 | 66 | + |
| ND1 | 11638 | 12561 | 924 | − |
| tRNAL1 | 12581 | 12645 | 65 | − |
| rRNAL | 12604 | 13979 | 1376 | − |
| tRNAV | 13966 | 14037 | 72 | − |
| rRNAS | **14037** | 14820 | 784 | − |
