# Supplementary material for: Long-read based assembly and synteny analysis of a reference Drosophila subobscura genome reveals signatures of structural evolution driven by inversions recombination-suppression effects
Source: BMC Genomics. 2019 Mar 18;20:223. doi: 10.1186/s12864-019-5590-8 (PMC6423853; doi:10.1186/s12864-019-5590-8)
Supplement: Supplementary file 5 — Table S4. Repetitive content of the D. subobscura genome. (DOCX 43 kb) [file 12864_2019_5590_MOESM5_ESM.docx]

**Table S4.** Repetitive content of the *D. subobscura* genome.

| **Class** | **No. of copies** | **Length (bp)** | **% of genome^1^** |
| --- | --- | --- | --- |
| Retrotransposon |  |  |  |
| SINE | 168 | 14,783 | 0.01% |
| LINE | 9,080 | 2,760,714 | 2.14% |
| LTR | 7,036 | 2,317,534 | 1.79% |
| DNA TEs | 25,953 | 4,853,394 | 3.76% |
| P | 3,778 | 409,099 | 0.32% |
| CMC-EnSpm | 2,475 | 264,762 | 0.20% |
| Tc1/Mariner | 1,190 | 423,231 | 0.33% |
| hAT | 3,369 | 684,490 | 0.53% |
| T2/Kolobok | 981 | 93,683 | 0.07% |
| Helitrons | 6,490 | 1,817,722 | 1.41% |
| Maverick | 1,295 | 392,702 | 0.30% |
| Other | 6,375 | 767,705 | 0.59% |
|  |  |  |  |
| Simple repeat | 134,876 | 5,313,023 | 4.13% |
| Low complexity | 15,271 | 723,001 | 0.56% |
| Satellites |  |  |  |
| SGM | 5,181 | 2,298,546 | 1.78% |
| Sat290 | 637 | 103,534 | 0.08% |
| Other | 528 | 42,912 | 0.03% |
| Unclassified | 341 | 86,020 | 0.07% |
| Other | 2 | 367 | 0.0003% |
| Total | 199,073 | 18,531,828 | 14.34% |

1. Percents based on a 129,236,726 bp genome.
