# Supplementary material for: Long-read based assembly and synteny analysis of a reference Drosophila subobscura genome reveals signatures of structural evolution driven by inversions recombination-suppression effects
Source: BMC Genomics. 2019 Mar 18;20:223. doi: 10.1186/s12864-019-5590-8 (PMC6423853; doi:10.1186/s12864-019-5590-8)
Supplement: Supplementary file 7 — Table S5. Optimal CAFE model selection for the evolution of gene family size along the 14 Drosophila ultrametric tree in Figures S3-S4. Shown are the four assayed increasingly complex models, including the 1-λ and 3-λ models, and the 5-λ model without and with global assembling error term (ε); and their corresponding parameter estimates, including global (λG), slow (λS), medium (λM), fast (λF), D. subobscura (λDs) and D. guanche (λDg) lambdas, and global error, and maximum-likelihood scores (−lnL). (DOCX 42 kb) [file 12864_2019_5590_MOESM7_ESM.docx]

**Table S5**. Optimal CAFE model selection for the evolution of gene family size along the 14 *Drosophila* ultrametric tree in Figures S3-S4. Shown are the four assayed increasingly complex models, including the 1-λ and 3-λ models, and the 5-λ model without and with global assembling error term (ε); and their corresponding parameter estimates, including global (λ_G_), slow (λ_S_), medium (λ_M_), fast (λ_F_), *D. subobscura* (λ_Ds_) and *D. guanche* (λ_Dg_) lambdas, and global error, and maximum-likelihood scores (-lnL).

| Model | λ_G_ | λ_S_ | λ_M_ | λ_F_ | λ_Ds_ | λ_Dg_ | ε | -lnL |
| --- | --- | --- | --- | --- | --- | --- | --- | --- |
|  |  |  |  |  |  |  |  |  |
| 1-λ | 0.0027 |  |  |  |  |  |  | 103,172.40 |
| 3-λ |  | 0.0009 | 0.0024 | 0.0218 |  |  |  | 88,126.00 |
| 5-λ |  | 0.0009 | 0.0024 | 0.0216 | 0.0257 | 0.0191 |  | 88,104.66 |
| 5-λ + ε |  | 0.0007 | 0.0018 | 0.0124 | 0.0197 | 0.0112 | 0.0747 | 88,008.67 |
