# Supplementary material for: Long-read based assembly and synteny analysis of a reference Drosophila subobscura genome reveals signatures of structural evolution driven by inversions recombination-suppression effects
Source: BMC Genomics. 2019 Mar 18;20:223. doi: 10.1186/s12864-019-5590-8 (PMC6423853; doi:10.1186/s12864-019-5590-8)
Supplement: Supplementary file 10 — Table S6. Over represented GO Terms among CAFE significantly expanded gene families in D. subobscura inferred using one-sided Fisher exact test (FDR < 0.001) implemented in Blast2Go (BP: Biological Process; MF: Molecular Function; CC: Cellular Component). (DOCX 46 kb) [file 12864_2019_5590_MOESM10_ESM.docx]

**Table S6.** Over represented GO Terms among CAFE significantly expanded gene families in *D. subobscura* inferred using one−sided Fisher exact test (FDR < 0.001) implemented in Blast2Go (BP: Biological Process; MF: Molecular Function; CC: Cellular Component).

| GO ID | GO name | GO category | FDR |
| --- | --- | --- | --- |
| GO:0001883 | Purine nucleoside binding | MF | 4.55E-20 |
| GO:0001745 | Compound eye morphogenesis | BP | 5.42E-04 |
| GO:0042278 | Purine nucleoside metabolic process | BP | 3.58E-31 |
| GO:0006338 | Chromatin remodeling | BP | 3.20E-16 |
| GO:0005703 | Polytene chromosome puff | CC | 7.10E-18 |
| GO:0003682 | Chromatin binding | MF | 1.58E-09 |
| GO:0000124 | SAGA complex | CC | 1.50E-27 |
| GO:0008134 | Transcription factor binding | MF | 1.59E-05 |
| GO:0005730 | Nucleolus | CC | 1.46E-06 |
| GO:0007362 | Terminal region determination | BP | 4.95E-19 |
| GO:0010485 | H4 histone acetyltransferase activity | MF | 1.52E-26 |
| GO:0005671 | Ada2/Gcn5/Ada3 transcription activator complex | CC | 2.63E-22 |
| GO:0048515 | Spermatid differentiation | BP | 5.39E-06 |
| GO:0016604 | Nuclear body | CC | 6.34E-12 |
| GO:0007478 | Leg disc morphogenesis | BP | 4.46E-04 |
| GO:0060828 | Regulation of canonical Wnt signaling pathway | BP | 9.00E-04 |
| GO:1901605 | Alpha-amino acid metabolic process | BP | 7.60E-04 |
| GO:0051568 | Histone H3-K4 methylation | BP | 2.64E-07 |
| GO:0005201 | Extracellular matrix structural constituent | MF | 2.50E-06 |
| GO:0008347 | Glial cell migration | BP | 7.84E-06 |
| GO:0006352 | DNA-templated transcription, initiation | BP | 2.34E-04 |
| GO:0003713 | Transcription coactivator activity | MF | 3.96E-04 |
| GO:0032968 | Positive regulation of transcription elongation from RNA polymerase II promoter | BP | 4.78E-06 |
| GO:0040040 | Thermosensory behavior | BP | 1.96E-05 |
| GO:0048864 | Stem cell development | BP | 4.03E-04 |
| GO:0043971 | Histone H3-K18 acetylation | BP | 4.44E-12 |
| GO:1990226 | Histone methyltransferase binding | MF | 4.44E-12 |
| GO:0043993 | Histone acetyltransferase activity (H3-K18 specific) | MF | 4.44E-12 |
| GO:0044017 | Histone acetyltransferase activity (H3-K27 specific) | MF | 4.44E-12 |
| GO:0043982 | Histone H4-K8 acetylation | BP | 1.60E-11 |
| GO:0043983 | Histone H4-K12 acetylation | BP | 4.89E-11 |
| GO:0043974 | Histone H3-K27 acetylation | BP | 1.41E-09 |
| GO:0032922 | Circadian regulation of gene expression | BP | 2.42E-08 |
| GO:0000076 | DNA replication checkpoint | BP | 1.35E-07 |
| GO:0035023 | Regulation of Rho protein signal transduction | BP | 4.71E-05 |
| GO:0007464 | R3/R4 cell fate commitment | BP | 8.61E-05 |
| GO:0004777 | Succinate-semialdehyde dehydrogenase (NAD+) activity | MF | 3.56E-11 |
| GO:0005604 | Basement membrane | CC | 3.48E-04 |
| GO:0008266 | Poly(U) RNA binding | MF | 8.22E-05 |
| GO:0005844 | Polysome | CC | 8.22E-05 |
| GO:0005125 | Cytokine activity | MF | 1.57E-04 |
| GO:0046426 | Negative regulation of JAK-STAT cascade | BP | 9.57E-04 |
| GO:0030350 | Iron-responsive element binding | MF | 4.52E-07 |
| GO:0003994 | Aconitate hydratase activity | MF | 7.55E-06 |
| GO:0045252 | Oxoglutarate dehydrogenase complex | CC | 2.78E-04 |
| GO:0016624 | Oxidoreductase activity, acting on the aldehyde or oxo group of donors, disulfide as acceptor | MF | 7.49E-04 |
| GO:0006750 | Glutathione biosynthetic process | BP | 7.18E-04 |
| GO:0016846 | Carbon-sulfur lyase activity | MF | 7.18E-04 |
| GO:1900026 | Positive regulation of substrate adhesion-dependent cell spreading | BP | 5.93E-04 |
| GO:0035386 | Regulation of Roundabout signaling pathway | BP | 5.93E-04 |
| GO:0070899 | Mitochondrial tRNA wobble uridine modification | BP | 5.93E-04 |
| GO:0004029 | Aldehyde dehydrogenase (NAD) activity | MF | 5.93E-04 |
