# Supplementary material for: Long-read based assembly and synteny analysis of a reference Drosophila subobscura genome reveals signatures of structural evolution driven by inversions recombination-suppression effects
Source: BMC Genomics. 2019 Mar 18;20:223. doi: 10.1186/s12864-019-5590-8 (PMC6423853; doi:10.1186/s12864-019-5590-8)
Supplement: Supplementary file 11 — Table S7. Over represented GO Terms among CAFE significantly contracted gene families in D. subobscura inferred using one-sided Fisher exact test (FDR < 0.001) implemented in Blast2Go (BP: Biological Process; MF: Molecular Function; CC: Cellular Component). (DOCX 47 kb) [file 12864_2019_5590_MOESM11_ESM.docx]

**Table S7.** Over represented GO Terms among CAFE significantly contracted gene families in *D. subobscura* inferred using one−sided Fisher exact test (FDR < 0.001) implemented in Blast2Go (BP: Biological Process; MF: Molecular Function; CC: Cellular Component).

| GO ID | GO name | GO category | FDR |
| --- | --- | --- | --- |
| GO:0007605 | Sensory perception of sound | BP | 2.61E-20 |
| GO:0010114 | Response to red light | BP | 4.58E-20 |
| GO:0060086 | Circadian temperature homeostasis | BP | 1.08E-19 |
| GO:0004714 | Transmembrane receptor protein tyrosine kinase activity | MF | 8.37E-18 |
| GO:0043153 | Entrainment of circadian clock by photoperiod | BP | 1.04E-17 |
| GO:0035271 | Ring gland development | BP | 1.40E-17 |
| GO:0016061 | Regulation of light-activated channel activity | BP | 2.62E-17 |
| GO:0031489 | Myosin V binding | MF | 2.62E-17 |
| GO:2001259 | Positive regulation of cation channel activity | BP | 4.75E-16 |
| GO:0070855 | Myosin VI head/neck binding | MF | 1.15E-15 |
| GO:0004715 | Non-membrane spanning protein tyrosine kinase activity | MF | 1.15E-15 |
| GO:0031476 | Myosin VI complex | CC | 4.14E-14 |
| GO:0031475 | Myosin V complex | CC | 1.64E-13 |
| GO:0016060 | Metarhodopsin inactivation | BP | 5.09E-13 |
| GO:0097431 | Mitotic spindle pole | CC | 1.13E-12 |
| GO:0070865 | Investment cone | CC | 1.13E-12 |
| GO:0051383 | Kinetochore organization | BP | 1.39E-11 |
| GO:0016062 | Adaptation of rhodopsin mediated signaling | BP | 1.39E-11 |
| GO:0072499 | Photoreceptor cell axon guidance | BP | 2.18E-11 |
| GO:0010977 | Negative regulation of neuron projection development | BP | 4.77E-11 |
| GO:0038083 | Peptidyl-tyrosine autophosphorylation | BP | 1.25E-10 |
| GO:0031935 | Regulation of chromatin silencing | BP | 1.65E-10 |
| GO:0005876 | Spindle microtubule | CC | 3.26E-10 |
| GO:0001752 | Compound eye photoreceptor fate commitment | BP | 6.67E-10 |
| GO:0003705 | Transcription factor activity, RNA polymerase II distal enhancer sequence-specific binding | MF | 9.93E-10 |
| GO:0010705 | Meiotic DNA double-strand break processing involved in reciprocal meiotic recombination | BP | 1.46E-09 |
| GO:0010780 | Meiotic DNA double-strand break formation involved in reciprocal meiotic recombination | BP | 1.46E-09 |
| GO:0046716 | Muscle cell cellular homeostasis | BP | 2.30E-09 |
| GO:0030496 | Midbody | CC | 2.47E-09 |
| GO:0008514 | Organic anion transmembrane transporter activity | MF | 3.20E-09 |
| GO:0005814 | Centriole | CC | 5.77E-09 |
| GO:0005887 | Integral component of plasma membrane | CC | 9.94E-09 |
| GO:0045316 | Negative regulation of compound eye photoreceptor development | BP | 1.14E-08 |
| GO:0007099 | Centriole replication | BP | 3.37E-08 |
| GO:0015711 | Organic anion transport | BP | 3.37E-08 |
| GO:0042052 | Rhabdomere development | BP | 3.38E-08 |
| GO:0043035 | Chromatin insulator sequence binding | MF | 3.50E-08 |
| GO:0030048 | Actin filament-based movement | BP | 5.56E-08 |
| GO:0070868 | Heterochromatin organization involved in chromatin silencing | BP | 7.02E-08 |
| GO:0000792 | Heterochromatin | CC | 8.11E-08 |
| GO:0031234 | Extrinsic component of cytoplasmic side of plasma membrane | CC | 2.32E-07 |
| GO:0007169 | Transmembrane receptor protein tyrosine kinase signaling pathway | BP | 5.76E-07 |
| GO:0045944 | Positive regulation of transcription by RNA polymerase II | BP | 6.38E-07 |
| GO:0007155 | Cell adhesion | BP | 6.73E-07 |
| GO:0061332 | Malpighian tubule bud morphogenesis | BP | 1.07E-06 |
| GO:0042127 | Regulation of cell proliferation | BP | 2.21E-06 |
| GO:0030178 | Negative regulation of Wnt signaling pathway | BP | 3.43E-06 |
| GO:0045931 | Positive regulation of mitotic cell cycle | BP | 3.97E-06 |
| GO:0007390 | Germ-band shortening | BP | 4.22E-06 |
| GO:0005813 | Centrosome | CC | 4.37E-06 |
| GO:0005326 | Neurotransmitter transporter activity | MF | 5.43E-06 |
| GO:0035071 | Salivary gland cell autophagic cell death | BP | 6.35E-06 |
| GO:0016028 | Rhabdomere | CC | 6.83E-06 |
| GO:0035075 | Response to ecdysone | BP | 7.24E-06 |
| GO:0046960 | Sensitization | BP | 1.75E-05 |
| GO:0015695 | Organic cation transport | BP | 2.15E-05 |
| GO:0007485 | Imaginal disc-derived male genitalia development | BP | 2.82E-05 |
| GO:0000788 | Nuclear nucleosome | CC | 2.82E-05 |
| GO:0035074 | Pupation | BP | 3.38E-05 |
| GO:0007402 | Ganglion mother cell fate determination | BP | 3.38E-05 |
| GO:0090303 | Positive regulation of wound healing | BP | 3.38E-05 |
| GO:0035230 | Cytoneme | CC | 6.32E-05 |
| GO:0007424 | Open tracheal system development | BP | 6.32E-05 |
| GO:0001078 | Proximal promoter DNA-binding transcription repressor activity, RNA polymerase II-specific | MF | 9.79E-05 |
| GO:0005509 | Calcium ion binding | MF | 1.48E-04 |
| GO:0043065 | Positive regulation of apoptotic process | BP | 2.70E-04 |
| GO:0007476 | Imaginal disc-derived wing morphogenesis | BP | 3.53E-04 |
| GO:0045087 | Innate immune response | BP | 4.73E-04 |
| GO:0000166 | Nucleotide binding | MF | 4.86E-04 |
| GO:0035172 | Hemocyte proliferation | BP | 6.10E-04 |
| GO:0000187 | Activation of MAPK activity | BP | 6.34E-04 |
| GO:0000978 | RNA pol II proximal promoter sequence-specific DNA binding | MF | 6.46E-04 |
| GO:0021579 | Medulla oblongata morphogenesis | BP | 7.59E-04 |
| GO:1902843 | Positive regulation of netrin-activated signaling pathway | BP | 7.59E-04 |
| GO:0007267 | Cell-cell signaling | BP | 8.31E-04 |
| GO:0007517 | Muscle organ development | BP | 8.70E-04 |
| GO:0006334 | Nucleosome assembly | BP | 8.80E-04 |
