# Supplementary material for: Long-read based assembly and synteny analysis of a reference Drosophila subobscura genome reveals signatures of structural evolution driven by inversions recombination-suppression effects
Source: BMC Genomics. 2019 Mar 18;20:223. doi: 10.1186/s12864-019-5590-8 (PMC6423853; doi:10.1186/s12864-019-5590-8)
Supplement: Supplementary file 18 — Table S8. Number of syntenic blocks between D. subobscura and increasingly distant relatives. (DOCX 41 kb) [file 12864_2019_5590_MOESM18_ESM.docx]

**Table S8.** Number of syntenic blocks between *D. subobscura* and increasingly distant relatives.

|  |  |  | A | J | U | E | O | Total |
| --- | --- | --- | --- | --- | --- | --- | --- | --- |
| *D. subobscura* | × | *D. guanche* | 12 | 3 | 4 | 6 | 6 | 31 |
|  | × | *D. pseudoobscura* | 90 | 66 | 56 | 59 | 62 | 333 |
|  | × | *D. melanogaster* | 125 | 100 | 87 | 115 | 113 | 540 |
