# Supplementary material for: Long-read based assembly and synteny analysis of a reference Drosophila subobscura genome reveals signatures of structural evolution driven by inversions recombination-suppression effects
Source: BMC Genomics. 2019 Mar 18;20:223. doi: 10.1186/s12864-019-5590-8 (PMC6423853; doi:10.1186/s12864-019-5590-8)
Supplement: Supplementary file 19 — Table S9. Average size of the syntenic block (in Mb) between D. subobscura and increasingly distant relatives. (DOCX 41 kb) [file 12864_2019_5590_MOESM19_ESM.docx]

**Table S9.** Average size of the syntenic block (in Mb) between *D. subobscura* and increasingly distant relatives.

|  |  |  | A | J | U | E | O | Total |
| --- | --- | --- | --- | --- | --- | --- | --- | --- |
| *D. subobscura* | × | *D. guanche* | 1.364 | 7.826 | 4.155 | 3.578 | 4.780 | 3.952 |
|  | × | *D. pseudoobscura* | 0.224 | 0.333 | 0.439 | 0.333 | 0.462 | 0.345 |
|  | × | *D. melanogaster* | 0.172 | 0.226 | 0.284 | 0.175 | 0.264 | 0.220 |
