# Supplementary material for: Long-read based assembly and synteny analysis of a reference Drosophila subobscura genome reveals signatures of structural evolution driven by inversions recombination-suppression effects
Source: BMC Genomics. 2019 Mar 18;20:223. doi: 10.1186/s12864-019-5590-8 (PMC6423853; doi:10.1186/s12864-019-5590-8)
Supplement: Supplementary file 20 — Figure S11. Schematic of the strategy used for inversion breakpoint detection. From top to bottom: shown are (a) two noninverted (SB1 and SB3; pink) and one inverted (SB2; green) hypothetical SyMAP synteny blocks between two taxa (1 and 2). The regions flanking the points of broken synteny (vertical dotted lines) are labelled A-D correspondingly; (b) BLASTing regions AB and CD from taxon 1 against the genome of taxon 2 each produces two hits (c) at opposite ends of the inverted synteny block with associated overhangs; (d) steps b-c are repeated using taxon 2 for the BLAST queries to test for reciprocal consistency (see main text for more detail). (PDF 97 kb) [file 12864_2019_5590_MOESM20_ESM.pdf]

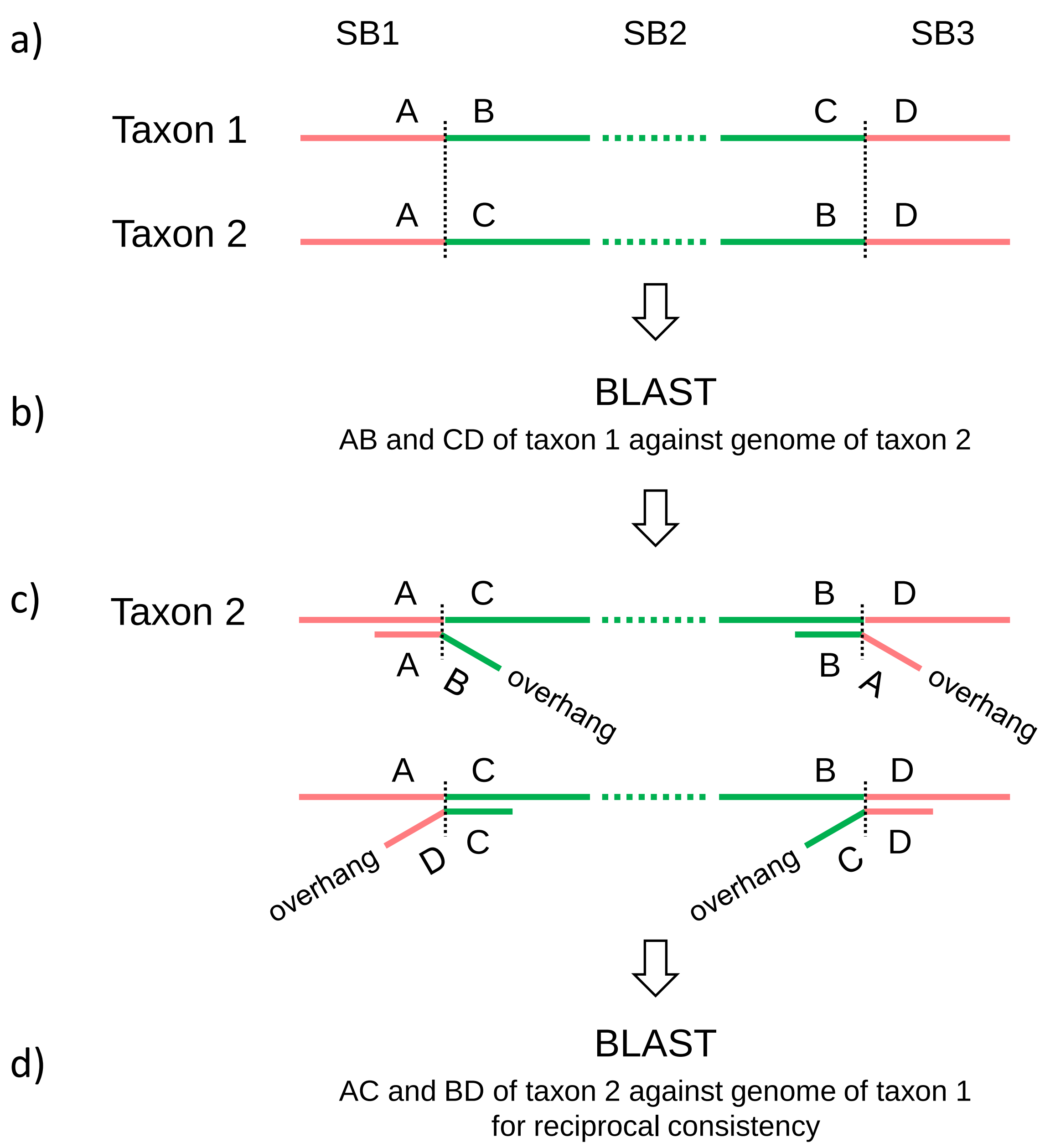
a)
SB1
SB2
SB3
Taxon 1
A B C D
Taxon 2
A C B D
b)
BLAST
AB and CD of taxon 1 against genome of taxon 2
c)
Taxon 2
A C B D
A B overhang
B A overhang
A C B D
overhang D C
overhang C D
d)
BLAST
AC and BD of taxon 2 against genome of taxon 1
for reciprocal consistency
